# Supplementary material for: Binational cohort study comparing the management and outcomes of pregnant women with a BMI >50–59.9 kg/m2 and those with a BMI ≥60 kg/m2
Source: BMJ Open. 2018 Aug 10;8(8):e021055. doi: 10.1136/bmjopen-2017-021055 (PMC6089316; doi:10.1136/bmjopen-2017-021055)
Supplement: Supplementary data [file bmjopen-2017-021055supp001.pdf]

Supplementary table 1. Risk factors and administration of postnatal thromboprophylaxis in UK population: Green Top guideline no. 37a. Royal College of Obstetrics and Gynaecology

	Did not receive thromboprophylaxis n (%)		Received postnatal thromboprophylaxis n (%)	
Only risk factor BMI ≥40	7	(4.2)	3	(0.7)
Two or more risk factors	160	(95.8)	432	(99.3)
Should have received thromboprophylaxis**	**167**	**(27.7)**	435	(72.2)

*These included: caesarean section, age ≥35, infection, parity ≥3, smoker, preeclampsia, caesarean section, multiple births and stillbirth (Other risk factors were in the RCOG guideline that were not available in this dataset).

**Row percentage

Supplementary table 2. Administration for postnatal thromboprophylaxis and criteria for guideline in Australia

	Did not receive thromboprophylaxis n (%)		Received thromboprophylaxis n (%)	
Meet the guideline criteria	109	(97.3)	192	(97.7)
Did not meet the guideline criteria	3	(2.7)	4	(2.0)
Should have received thromboprophylaxis***	**109**	**(36.2)**	192	(63.8)

*Major risk factors included: caesarean section, preeclampsia, infection and BMI ≥35kg/m^2

**Minor risk factors included: Age > 35 years, smoker, post-partum haemorrhage and parity ≥3 (Other risk factors were in the guideline that were not available in this dataset).

***Row percentage

Summary for indication for post-partum prophylactic anticoagulation for South Australian Perinatal Practice Guidelines thromboprophylaxis and thromboembolic disease in pregnancy by South Australian Maternal & Neonatal Clinical Network

-Emergency caesarean section OR 2 or more major risk factors

-At least one major and 2 or more minor risk factors
