# Supplementary material for: Binational cohort study comparing the management and outcomes of pregnant women with a BMI >50–59.9 kg/m2 and those with a BMI ≥60 kg/m2
Source: BMJ Open. 2018 Aug 10;8(8):e021055. doi: 10.1136/bmjopen-2017-021055 (PMC6089316; doi:10.1136/bmjopen-2017-021055)
Supplement: Author's manuscript [file bmjopen-2017-021055.draft_revisions.pdf]

BMJ Open

BMJ Open is committed to open peer review. As part of this commitment we make the peer review history of every article we publish publicly available.

When an article is published we post the peer reviewers' comments and the authors' responses online. We also post the versions of the paper that were used during peer review. These are the versions that the peer review comments apply to.

The versions of the paper that follow are the versions that were submitted during the peer review process. They are not the versions of record or the final published versions. They should not be cited or distributed as the published version of this manuscript.

BMJ Open is an open access journal and the full, final, typeset and author-corrected version of record of the manuscript is available on our site with no access controls, subscription charges or pay-per-view fees (http://bmjopen.bmj.com).

If you have any questions on BMJ Open's open peer review process please email info.bmjopen@bmj.com

A bi-national cohort study comparing the management and outcomes of pregnancy women with a BMI>50-<60kg/m2 and those with a BMI ≥60kg/m2

Journal:	*BMJ Open*
Manuscript ID	bmjopen-2017-021055
Article Type:	Research
Date Submitted by the Author:	08-Dec-2017
Complete List of Authors:	McCall, Stephen; University of Oxford Said Business School, National Perinatal Epidemiology Unit, Nuffield Department of Population Health<br>Li, Zhuoyang; University of Technology Sydney Faculty of Health, Australian Centre for Public and Population Health Research<br>Kurinczuk, Jenny; University of Oxford, National Perinatal Epidemiology Unit<br>Sullivan, Elizabeth; University of Technology Sydney Faculty of Health, Australian Centre for Public and Population Health Research<br>Knight, Marian; National Perinatal Epidemiology Unit
Keywords:	Obesity, OBSTETRICS, obesity in pregnancy

SCHOLARONE™
Manuscripts

For peer review only

A bi-national cohort study comparing the management and outcomes of pregnancy women with a BMI>50-<60kg/m^2 and those with a BMI ≥60kg/m^2

Running title: The management, maternal and perinatal outcomes of women with a BMI ≥60kg/m^2

Stephen J. McCall[1]; Zhuoyang Li[2], Jennifer J. Kurinczuk[1], Elizabeth Sullivan[2]*, Marian Knight[1]*

[1] National Perinatal Epidemiology Unit (NPEU), Nuffield Department of Population Health, University of Oxford, Oxford, UK

[2]The Australian Centre for Public and Population Health Research, University of Technology Sydney, Sydney, Australia

*Joint senior authors

Address for correspondence: Stephen McCall, National Perinatal Epidemiology Unit, Nuffield Department of Population Health, University of Oxford, Richard Doll Building, Old Road Campus, Oxford, OX3 7LF, UK.

Email: Stephen.mccall@npeu.ox.ac.uk

Word count: 2313

For peer review only

Abstract

Objectives: To compare the management, maternal and perinatal outcomes of women with a BMI ≥60kg/m^2 with women with a BMI >50-<60kg/m^2.

Design: International collaborative cohort study

Setting: Bi-national study in the UK and Australia

Participants: UK: all pregnant women and Australia: women gave birth (birthweight ≥400g or gestation ≥20 weeks

Methods: Data from the Australasian Maternity Outcomes Surveillance System and UK Obstetric Surveillance System. Management, maternal and infant outcomes were compared between women with BMI ≥60kg/m^2 and women with a BMI >50-<60kg/m^2, using unconditional logistic regression.

Results: The sociodemographic characteristics and previous medical histories were similar between the 111 women with a BMI ≥60kg/m^2 and the 821 women with a BMI >50-<60kg/m^2. Women with a BMI ≥60kg/m^2 had higher odds of thromboprophylaxis usage in both the antenatal (24% vs 12%; OR:2.25, 95%CI:1.39-3.64) and postpartum periods (78% vs 66%; OR:1.6, 95CI:1.04-2.70). Women with BMI ≥60kg/m^2 had nearly double the odds of preeclampsia (adjusted OR:1.83 (95%CI:1.01-3.30)). No other maternal or perinatal outcomes were statistically significantly different. Severe adverse outcomes such as perinatal death were uncommon in both groups thus limiting the power of these comparisons. The rate of perinatal deaths was 18 per 1000 births for those with BMI≥60 kg/m^2; 12.1 per 1000 births for those with BMI>50-<60 kg/m^2; those with BMI≥60 kg/m^2 had a non-significant increased odds of perinatal death (unadjusted OR:1.46, 95% CI:0.31-6.73).

For peer review only - http://bmjopen.bmj.com/site/about/guidelines.xhtml

Conclusions: The preeclampsia result suggests that weight reduction of any amount prior to pregnancy could reduce poor outcomes even if women remain extremely obese. Women are managed differently on the basis of BMI even at this extreme as shown by thromboprophylaxis.

Strengths

- Population based study examining extreme obesity using national data from the UK and Australia.
- International collaborative studies allow the examination of rare exposures.

Limitations

-This study lacked the power to examine many maternal and perinatal outcomes despite having data from two national studies

-Some outcomes were not comparable between Australia and the UK so could not be explored.

For peer review only

Introduction

Increasing rates of obesity in the general population are associated with an increasing trend towards obesity in pregnancy (Heslehurst et al., 2010). Within the general population, the largest increases in obesity have been in the highest BMI groups (Sturm, 2007), and this is also true for extreme obesity in pregnancy (Kim et al., 2007). This is problematic as maternal obesity is a risk factor for a number of pregnancy related complications (Bhattacharya et al., 2007, Catalano and Ehrenberg, 2006).

There have been several studies investigating the prevalence, outcomes and managements of extreme obesity in pregnancy (BMI ≥50 kg/m^2) (Crane et al., 2013, Martin et al., 2014, Knight et al., 2010, Marshall et al., 2012). These have aimed to test whether there was a dose response relationship between increased BMI and complications of pregnancy. Within the extremely obese group (BMI ≥50 kg/m^2) women included have had a BMI ranging from ≥50 kg/m^2 to approximately 75 kg/m^2. Whilst it may be the case that the risks rise exponentially with BMI it is possible that above a certain BMI, the risks of maternal and perinatal complications as a result of obesity do not increase due to the competing risks of other comorbidities. This remains to be investigated, as current published data do not allow the division of women into the highest BMI groups.

Previous research pooling together international data on rare exposures in pregnancy has been limited due to heterogeneity of definitions, methods and populations (Knight et al., 2009). The obstetric surveillance systems in Australia and the UK were designed to be compatible with data collection using similar definitions with a view to pooling data. As a result, there are comparable data available to combine national

studies, providing a large enough sample compare two groups of women within a cohort of extremely obese women. This study aimed to compare the characteristics, management, maternal and perinatal outcomes of women at the extremes of obesity.

For peer review only

Methods

Study population and design

This study was an international population-based cohort study, using secondary analysis of two national cohort studies of extreme maternal obesity , which were undertaken in Australia and the United Kingdom (Sullivan et al., 2015, Knight et al., 2010). For the purposes of the analysis, the exposed cohort were those pregnant women who had a BMI ≥60 kg/m^2 and the unexposed comparison cohort were those with a BMI >50-<60 kg/m^2.

Anonymous data were prospectively collected using each of the national obstetric surveillance system the United Kingdom Obstetric Surveillance System (UKOSS) or Australasian Maternity Outcomes Surveillance System (AMOSS). The methods of each system have been described elsewhere in detail (Knight et al., 2005, Sullivan et al., 2015, McDonnell et al., 2015). UKOSS data were collected from all UK consultant-led obstetric units while AMOSS data were collected nationally from all hospitals with over 50 births per year in Australia.

Outcomes, management and potential covariates relevant to the research question were identified from the literature. On the basis of this, possible covariates and outcomes were identified in the respective UKOSS and AMOSS datasets. Each variable was mapped between the AMOSS and UKOSS datasets and an assessment of the comparability was made. On occasions, where the coding differed, harmonisation of the coding was devised and applied. This resulted in uniform values and labels of variables across both datasets. An assessment was made to determine whether the variables were measuring the same clinical phenotype in similar ways.

For peer review only - http://bmjopen.bmj.com/site/about/guidelines.xhtml

The covariates explored in the analysis were age, smoking status during pregnancy, previous pregnancy problems, pre-existing medical problems, pre-existing hypertension, parity and multiple pregnancies.

The missing data in similar datasets has been shown not to be missing at random; as a result multiple imputation was not considered appropriate (Lindquist et al., 2013). A missing category was this created for each variable to account for the missing data. Primarily, complete case analysis was used in the multivariable analysis and a sensitivity analysis including the missing categories was used to assess the impact of missing data on the point estimates.

The sample size was predetermined by the size of the existing studies; therefore the sample was fixed at 111 women who had a BMI ≥60 kg/m^2 and 821 women who had a BMI >50-<60kg/m^2. For the lowest frequency outcome (perinatal death), which had an incidence of 1.2% in the unexposed group, given the sample size the minimum odds ratio detectable as statistically significant with 80% power at the 5% significance level was 5.63. For the highest frequency outcome, which had an incidence of 66.4% (thromboprophylaxis postnatally) in the unexposed group, the minimum odds ratio detectable as statistically significant with 80% power at the 5% significance level was 1.99.

Statistical analysis

Descriptive analyses were undertaken using the Chi Square test or Wilcoxon rank sum test as appropriate. These analyses assessed whether there was a statistical difference in characteristics between those women who had a BMI ≥60 kg/m^2 and those with a BMI >50-59 kg/m^2.

For peer review only

Each outcome was individually modelled in a univariable analysis using unconditional logistic regression, with results presented as unadjusted odds ratios (uOR) with 95% confidence intervals (95% CI). The exposure variable in each model was extreme obesity BMI ≥60 kg/m^2. To account for clustering of infants within mothers' (multiple births) robust estimates of variance were calculated. Collinearity was assessed between all plausible linear associations prior to multivariable analysis, using Pearson's correlation coefficient.

Only outcomes that were statistically significant at the univariable level were included in the multivariable analysis. In the multivariable analysis, potential explanatory variables were sequentially added to the univariable model in a forward stepwise method with an examination of the results as each variable was added. A plausible explanatory variable was included in the final model if it was associated with the exposure and outcome (P-value for Wald test<0.05) or significantly improved the model fit assessed by likelihood ratio tests at the 5% significance level. Statistical analysis was completed using STATA V.13 (STATA CORP, Texas, USA).

A post hoc analysis was completed to assess the risk factors for a thrombotic events possessed by those who did not receive postnatal thromboprophylaxis. This was a country specific analysis using risk factors of thrombotic events which were identified from the RCOG and South Australian Maternal & Neonatal Clinical Network guidelines (Royal College of Obstetrics and Gynaecology (RCOG), 2015, South Australian Maternal & Neonatal Clinical Network., 2013).

For peer review only - http://bmjopen.bmj.com/site/about/guidelines.xhtml

Ethics committee approval

The Australian collaborators obtained approval for the study from the NSW Population and Health Services Research Ethics Committee and multiple Human Research Ethics Committees across Australia (Vaughan et al., 2012). Ethics committee approval for secondary analysis of anonymous UK data was not required.

For peer review only

Results

During the period September 2007-August 2008, 617 women with a BMI>50 kg/m^2 were identified through the UK Obstetric Surveillance System. Between January - October 2010, 315 women with a BMI>50 kg/m^2 were identified using the Australasian Maternity Outcomes Surveillance System. Overall there were 111 women with a BMI ≥60kg/m^2 and 821 women with a BMI >50-<60kg/m^2.

Women with a BMI ≥60kg/m^2 were slightly older, the sociodemographic characteristics and previous medical histories were otherwise similar the two groups of women (Table 1).

A high proportion in both groups experienced ultrasounds scanning problems (70.3% vs. 65.7%) although this was not statistically significant between the groups. Fewer women in both groups received antenatal thromboprophylaxis (24.3% BMI ≥60kg/m^2 and 12.3% >50-<60kg/m^2) compared to postnatal thromboprophylaxis (77.5% and 66.4%) (Table 2). Women with a BMI ≥60kg/m^2 had a significantly higher odds of preeclampsia (uOR: 1.91 (95%CI: 1.08-3.39)), and of receiving either thromboprophylaxis antenatally (uOR:2.25 (95%CI:1.39-3.64)) or postnatally (uOR: 1.68 (95%CI: 1.04-2.70)) compared to those with a BMI >50-<60kg/m^2 (Table 2). Supplementary tables 1 and 2 show that 27% and 32% of women should have received thromboprophylaxis postnatally in the UK and Australia as they had the relevant risk factors for it to be indicated , respectively.

Although not statistically significant, a higher proportion of women with a BMI ≥60kg/m^2 experienced other adverse outcomes other than preeclampsia/eclampsia. Preeclampsia/eclampsia was examined in a multivariable model. The presence of a BMI ≥60kg/m^2 was associated with a two-fold increase in the odds of having

For peer review only - http://bmjopen.bmj.com/site/about/guidelines.xhtml

preeclampsia/eclampsia (aOR:1.83 (95%CI: 1.01-3.30)) compared to those with a BMI >50-59kg/m^2, after adjusting for smoking status, pre-existing diabetes and parity. The results of the proxy variable model did not materially differ to those of the complete case analysis.

Severe adverse outcomes such as perinatal death were uncommon in both groups (n=2 (18 per 1000 births), BMI≥60kg/m^2 vs. n=10 (12 per 1000 births), BMI ≥ 50-59kg/m^2). There were no statistically significant differences in perinatal outcomes between the both obesity groups (see table 3).

For peer review only

Discussion

Main Findings

Compared with women with a BMI >50-59 women with a BMI≥60 kg/m^2 had an increased risk of preeclampsia/eclampsia, suggesting any weight reduction could reduce poor outcomes even if women remain extremely obese. There were very few statistically significant differences in outcomes between the two very high BMI groups. Nevertheless, the direction of effects favours the lower BMI group for most outcomes. Importantly, the perinatal mortality rate was higher in both groups compared with both the UK/Australian rate. Women are being managed differently on the basis of BMI even at this extreme as use of thromboprophylactic drugs varied between the two high BMI groups.

Strengths and limitations

Both prospective population based surveillance systems use a robust methodology, which reduces the risk of selection bias. Two national studies allowed the examination of women with a BMI ≥60 kg/m^2 in a high resource setting and thus overcomes some of the limitations of previous research which was limited by the number of women in the extreme ends of the BMI distribution. Nevertheless, despite pooling of national data the number of women in each group was still relatively small, which limited the study power, particularly when investigating rare outcomes.

Interpretation

One of the novel benefits of this multi-national study was the ability to examine a subset of the more extreme end of the spectrum of obesity. This demonstrated that women with a BMI>60 kg/m^2 had very similar characteristics and experienced similar

For peer review only - http://bmjopen.bmj.com/site/about/guidelines.xhtml

management compared to women with a BMI>50 -<60 kg/m^2. Interestingly, the BMI>60 kg/m^2 group had an increased risk of preeclampsia/eclampsia; which supports the hypothesis of a 'dose response' relationship between obesity and preeclampsia/eclampsia seen at lower BMIs (Bodnar et al., 2007) and super obesity (Marshall et al., 2012, Mbah et al., 2010) .

The BMI>60 kg/m^2 cohort had a higher proportion of perinatal deaths and stillbirths than the BMI >50-<60 kg/m^2 cohort, although these were not statistically significantly different possibly because of the small numbers involved. The absolute rate of perinatal death for the ≥60 kg/m^2 cohort was three times higher than the UK rate (5.6 per 1000 births) and 2.5 times higher than the Australian rate (7.3 per 1000); while the rate of perinatal mortality in the >50-<60 kg/m^2 cohort was just over twice that of the UK rate and was 1.5 times higher than the Australian perinatal mortality rate (Manktelow et al., 2017, AIHW: et al., 2016).

A previous study of extreme obesity that examine perinatal outcomes has suggested that there is a dose response relationship between BMI and perinatal outcomes (Marshall et al., 2012). The small sample size and relative rarity of adverse perinatal outcomes in this analysis did not allow the role of chance to be excluded for most outcomes even with pooling two national studies.

The results of this study show that the degree of relative obesity impacted on thromboprophylaxis practice. The Royal College of Obstetricians and Gynaecologists' guideline states that any women with a BMI >40kg/m^2 should be considered at intermediate risk of a thrombotic event and should be given at least 10 days of thromboprophylaxis postnatally (Royal College of Obstetrics and Gynaecology (RCOG), 2015). Within Australia there is regional variation in the

For peer review only - http://bmjopen.bmj.com/site/about/guidelines.xhtml

guidelines concerning BMI and postpartum thromboprophylaxis. The Queensland state guideline suggests that a woman must possess three or more risk factors (BMI >30 kg/m^2 being one of these risk factors) to be given low molecular weight heparin for 6 days postnatally, while the South Australian government and current expert opinion recommends that BMI ≥30 kg/m^2 plus one major risk factor for thromboembolism requires prophylactic anticoagulation for 5 days postpartum (Mclintock et al., 2012, South Australian Maternal & Neonatal Clinical Network., 2013). As there was large variation in practice between the two cohorts in this study this suggests that the guidelines are variably followed; suggesting more implementation work within clinical setting is needed to help these guidelines be to followed. Nevertheless, the results show that BMI has an important impact on clinical decisions concerning the administration of thromboprophylaxis postnatally.

Importantly, approximately 75% had postnatal thromboprophylaxis which is smaller than expected considering this was an extremely obese population. Nearly a third of women in both countries had the appropriate risk factors to indicate the use of thromboprophylaxis postnatally. This highlights an important area for improvement of clinical practice to prevent a potentially fatal thrombotic event.

Interestingly, there were no thrombotic events in the BMI ≥60 kg/m^2 group, which was the group in which the larger proportion of women received thromboprophylaxis although again these are very rare events. Previous studies have shown that BMI is a strong risk factor of thrombotic events (Larsen et al., 2007, Knight, 2008) and the risk is amplified in those who have a high BMI and were immobilized (Jacobsen et al., 2008).

For peer review only - http://bmjopen.bmj.com/site/about/guidelines.xhtml

Conclusions

There were very few statistically significant differences in outcomes between these two high BMI groups. However, the direction of effect favours the lower BMI group for most outcomes and a type II error cannot be excluded given the small number of outcomes. The preeclampsia risk in the higher BMI group and the direction of effect in other outcomes suggests that any weight reduction prior to pregnancy could reduce poor outcomes even if women remain extremely obese. Women are clearly being managed differently on the basis of BMI even at this extreme as shown by the thromboprophylaxis data.

Acknowledgements: Dr Manisha Nair and Dr Bryn Kemp for their advice on the project.

Disclosures of interest: None

Contribution to authorship:

Funding: SM is funded by the Nuffield Department of Population Health and Medical Research Council (MRC) training grant MR/K501256/1. Marian Knight is funded by an NIHR Research Professorship. The views expressed in this publication are those of the author(s), and not necessarily those of the MRC, the NHIR or the Department of Health. The funder had no role in the study design, data collection and analysis, decision to publish, or preparation of the article. The National Health and Medical Research Council Project Grant (Application 510298) for funding The Australian Maternity Outcomes Surveillance System: Improving safety and quality of maternity care in Australia (AMOSS) from 2008–2012.

Author contribution: MK, JK, ES conceived the study. MK, JK, ES and SM designed the study. ZL extracted the data. SM analysed the data and wrote the first draft. All authors interpreted the data and edited the manuscript.

For peer review only

Tables list

Table 1. Sociodemographic characteristics and previous medical problems in women with BMI ≥ $60kg/m^2$ and comparison women (BMI >50-<$60kg/m^2$).

Table 2. Maternal outcomes and management in women with BMI ≥ $60kg/m^2$ and comparison women (BMI >50-<$60kg/m^2$).

Table 3. Perinatal outcomes in women with BMI ≥ $60kg/m^2$ and comparison women (BMI >50-<$60kg/m^2$).

For peer review only

References

AIHW:, MONK, A., HARRIS, K., DONNOLLEY, N., HILDER, L., HUMPHREY, M., GORDON, A. & CHAMBERS, G. 2016. Perinatal deaths in Australia, 1993–2012. Perinatal deaths. Canberra: AIHW.

BHATTACHARYA, S., CAMPBELL, D. M., LISTON, W. A. & BHATTACHARYA, S. 2007. Effect of body mass index on pregnancy outcomes in nulliparous women delivering singleton babies. *BMC public Health,* 7**,** 168.

BODNAR, L. M., CATOV, J. M., KLEBANOFF, M. A., NESS, R. B. & ROBERTS, J. M. 2007. Prepregnancy body mass index and the occurrence of severe hypertensive disorders of pregnancy. *Epidemiology,* 18**,** 234-239.

CATALANO, P. M. & EHRENBERG, H. M. 2006. Review article: The short- and long-term implications of maternal obesity on the mother and her offspring. *BJOG: An International Journal of Obstetrics & Gynaecology,* 113**,** 1126-1133.

CRANE, J. M., MURPHY, P., BURRAGE, L. & HUTCHENS, D. 2013. Maternal and perinatal outcomes of extreme obesity in pregnancy. *Journal of Obstetrics & Gynaecology Canada: JOGC,* 35**,** 606-11.

HESLEHURST, N., RANKIN, J., WILKINSON, J. R. & SUMMERBELL, C. D. 2010. A nationally representative study of maternal obesity in England, UK: Trends in incidence and demographic inequalities in 619 323 births, 1989-2007. *International Journal of Obesity,* 34**,** 420-428.

JACOBSEN, A. F., SKJELDESTAD, F. E. & SANDSET, P. M. 2008. Ante- and postnatal risk factors of venous thrombosis: a hospital-based case–control study. *Journal of Thrombosis and Haemostasis,* 6**,** 905-912.

KIM, S. Y., DIETZ, P. M., ENGLAND, L., MORROW, B. & CALLAGHAN, W. M. 2007. Trends in Pre□pregnancy Obesity in Nine States, 1993–2003. *Obesity,* 15**,** 986-993.

KNIGHT, M. 2008. Antenatal pulmonary embolism: risk factors, management and outcomes. *BJOG: An International Journal of Obstetrics & Gynaecology,* 115**,** 453-461.

KNIGHT, M., CALLAGHAN, W. M., BERG, C., ALEXANDER, S., BOUVIER-COLLE, M. H., FORD, J. B., JOSEPH, K. S., LEWIS, G., LISTON, R. M., ROBERTS, C. L., OATS, J. & WALKER, J. 2009. Trends in postpartum hemorrhage in high resource countries: a review and recommendations from the International Postpartum Hemorrhage Collaborative Group. *BMC Pregnancy & Childbirth,* 9**,** 55.

KNIGHT, M., KURINCZUK, J. J., SPARK, P., BROCKLEHURST, P. & SYSTEM, U. K. O. S. 2010. Extreme obesity in pregnancy in the United Kingdom. *Obstetrics & Gynecology,* 115**,** 989-97.

KNIGHT, M., KURINCZUK, J. J., TUFFNELL, D. & BROCKLEHURST, P. 2005. The UK Obstetric Surveillance System for rare disorders of pregnancy. *BJOG: An International Journal of Obstetrics and Gynaecology,* 112**,** 263-265.

LARSEN, T. B., SØRENSEN, H. T., GISLUM, M. & JOHNSEN, S. P. 2007. Maternal smoking, obesity, and risk of venous thromboembolism during pregnancy and the puerperium: A population-based nested case-control study. *Thrombosis Research,* 120**,** 505-509.

LINDQUIST, A., KNIGHT, M. & KURINCZUK, J. J. 2013. Variation in severe maternal morbidity according to socioeconomic position: a UK national case–control study. *BMJ open,* 3**,** e002742.

MANKTELOW, B., SMITH, L., PRUNET, C., SMITH, P., BOBY, T., HYMAN-TAYLOR, P., KURINCZUK, J., FIELD, D., E., D. & ON BEHALF OF THE MBRRACE-UK COLLABORATION 2017. *MBRRACE-UK Perinatal Mortality Surveillance Report: UK Perinatal Deaths for Births from January to December 2015*, Department of Health Sciences, University of Leicester.

MARSHALL, N. E., GUILD, C., CHENG, Y. W., CAUGHEY, A. B. & HALLORAN, D. R. 2012. Maternal superobesity and perinatal outcomes. *American Journal of Obstetrics & Gynecology,* 206**,** 417.e1-6.

MARTIN, A., KRISHNA, I., ELLIS, J., PACCIONE, R. & BADELL, M. 2014. Super obesity in pregnancy: difficulties in clinical management. *Journal of Perinatology,* 34**,** 495-502.

MBAH, A. K., KORNOSKY, J. L., KRISTENSEN, S., AUGUST, E. M., ALIO, A. P., MARTY, P. J., BELOGOLOVKIN, V., BRUDER, K. & SALIHU, H. M. 2010. Super-obesity and risk for early and late pre-eclampsia. *BJOG: An International Journal of Obstetrics and Gynaecology,* 117**,** 997-1003.

MCDONNELL, N., KNIGHT, M., PEEK, M. J., ELLWOOD, D., HOMER, C. S. E., MCLINTOCK, C., VAUGHAN, G., POLLOCK, W., LI, Z., JAVID, N. & SULLIVAN, E. 2015. Amniotic fluid embolism: an Australian-New Zealand population-based study. *BMC Pregnancy and Childbirth,* 15**,** 352.

MCLINTOCK, C., BRIGHTON, T., CHUNILAL, S., DEKKER, G., MCDONNELL, N., MCRAE, S., MULLER, P., TRAN, H., WALTERS, B. N. & YOUNG, L. 2012. Recommendations for the prevention of pregnancy□associated venous thromboembolism. *Australian and New Zealand Journal of Obstetrics and Gynaecology,* 52**,** 3-13.

ROYAL COLLEGE OF OBSTETRICS AND GYNAECOLOGY (RCOG) 2015. Reducing the Risk of Venous Thromboembolism during Pregnancy and the Puerperium. Green-top Guideline No. 37a. London: RCOG.

SOUTH AUSTRALIAN MATERNAL & NEONATAL CLINICAL NETWORK. 2013. South Australian Perinatal Practice Guidelines: thromboprophylaxis and thromboembolic disease in pregnancy. . Department of Health, Government of South Australia.

STURM, R. 2007. Increases in morbid obesity in the USA: 2000–2005. *Public Health,* 121**,** 492-496.

SULLIVAN, E. A., DICKINSON, J. E., VAUGHAN, G. A., PEEK, M. J., ELLWOOD, D., HOMER, C. S. E., KNIGHT, M., MCLINTOCK, C., WANG, A., POLLOCK, W., JACKSON PULVER, L., LI, Z., JAVID, N., DENNEY-WILSON, E. & CALLAWAY, L. 2015. Maternal super-obesity and perinatal outcomes in Australia: A national population-based cohort study. *BMC Pregnancy and Childbirth,* 15.

VAUGHAN, G., POLLOCK, W., PEEK, M. J., KNIGHT, M., ELLWOOD, D., HOMER, C. S., PULVER, L. J., MCLINTOCK, C., HO, M. T. & SULLIVAN, E. A. 2012. Ethical issues: The multi-centre low-risk ethics/governance review process and AMOSS. *Australian and New Zealand Journal of Obstetrics and Gynaecology,* 52**,** 195-203.

For peer review only - http://bmjopen.bmj.com/site/about/guidelines.xhtml

For peer review only

Table 1. Sociodemographic characteristics and previous medical problems in women with BMI ≥ 60kg/m^2 and comparison women (BMI >50-<60kg/m^2).

Characteristic		Number (%) of obese women BMI ≥ 60 (n=111)		Number (%) of women BMI>50 - 59 (n=821)		p-value
Sociodemographic characteristics						
Age	*Mean (Std)*	31.7	(5.51)	30.3	(5.67)	0.017
BMI at booking	*Median (IQR)*	61.7	(60-64.9)	52.3	(50.8-54.9)	<0.001
Max recorded BMI	*Median (IQR)*	62.9	(61-66.8)	52.7	(50.9-55.0)	<0.001
Smoking status	*Never/ex-smoker*	85	(76.6)	599	(73)	0.42
	Smoked during pregnancy	24	(21.6)	206	(25.1)	
	Missing	2	(1.8)	16	(1.9)	
Known previous medical history						
Previous pregnancy problems	*None*	41	(36.9)	273	(33.3)	0.713
	Yes	33	(29.7)	266	(32.4)	
	Not applicable	35	(31.5)	271	(33.0)	
	Missing	2	(1.8)	11	(1.3)	
Known cardiac disease	*None*	109	(98.2)	812	(98.9)	0.713
	Yes	2	(1.8)	5	(0.6)	
	Missing	0	(0)	4	(0.5)	
Known renal disease	*None*	110	(99.1)	809	(98.5)	0.174
	Yes	1	(0.9)	8	(1.0)	
	Missing	0	(0)	4	(0.5)	
Known mental health issues	*None*	99	(89.2)	756	(92.1)	0.219
	Yes	12	(10.8)	61	(7.4)	
	Missing	0	(0)	4	(0.5)	
Known asthma	*None*	98	(88.3)	720	(87.7)	0.961
	Yes	13	(11.7)	97	(11.8)	
	Missing	0	(0)	4	(0.5)	
Previous caesarean delivery	*None*	53	(47.7)	366	(44.6)	0.297
	Yes	22	(19.8)	181	(22.0)	
	Not applicable	35	(31.5)	271	(33.0)	
	Missing	1	(0.9)	3	(0.4)	
Parity	*Nulliparous*	35	(31.5)	271	(33)	0.803
	Multiparous	75	(67.6)	550	(67)	
	Missing	1	(0.9)	0	(0)	
Current pregnancy						

Multiple pregnancy	*No*	108	(97.3)	800	(97.4)	0.928
	Yes	3	(2.7)	21	(2.6)	
	Missing					
Known hypertension prior to pregnancy	*None*	103	(92.8)	767	(93.4)	0.693
	Yes	8	(7.2)	51	(6.2)	
	Missing	0	(0)	3	(0.4)	
Known pre-existing diabetes prior to pregnancy	*None*	101	(91.0)	757	(92.2)	0.657
	Yes	10	(9.0)	64	(7.8)	
	Missing	-	-	-	-	
Insulin dependent diabetes	*Yes*	4	(3.6)	17	(2.1)	0.663

For peer review only - http://bmjopen.bmj.com/site/about/guidelines.xhtml

Table 2. Maternal outcomes and management in women with BMI ≥ 60kg/m^2 and comparison women (BMI >50-<60kg/m^2).

		Number (%) of women BMI ≥ 60 (n=111)		Number (%) of women BMI>50-59 (n=821)		Unadjusted odds ratio	95% Confidence interval	P-value
Management								
Scan problems	*No*	30	(27)	228	(27.8)	1		
	Yes	78	(70.3)	539	(65.7)	1.10	(0.70-1.72)	0.678
	Missing	3	(2.7)	54	(6.6)			
Induced	*No*	59	(53.2)	405	(49.3)	1		
	Yes	40	(36)	303	(36.9)	0.91	(0.59-1.39)	0.652
	Missing	12	(10.8)	113	(13.8)			
Syntocinon	*No*	25	(22.5)	243	(29.6)	1		
	Yes	41	(36.9)	289	(35.2)	1.38	(0.82-2.33)	0.231
	Missing	45	(40.5)	289	(35.2)			
Caesarean delivery	*No*	48	(43.2)	398	(48.5)	1		.
	Yes	62	(55.9)	411	(50.1)	1.25	(0.84,1.87)	0.274
	Missing	1	(0.9)	12	(1.5)			
Thromboprophylaxis usage antenatal	*No*	84	(75.7)	706	(86)	1		
	Yes	27	(24.3)	101	(12.3)	2.25	(1.39-3.64)	0.001
	Missing	0	(0)	14	(1.7)			
Thromboprophylaxis post	*No*	24	(21.6)	255	(31.1)	1		
	Yes	86	(77.5)	545	(66.4)	1.68	(1.04-2.70)	0.033
	Missing	1	(0.9)	21	(2.6)			
Maternal Outcome								
Wound infection in those with caesarean	*No*	47	(42.3)	344	(41.9)	1		
	Yes	14	(12.6)	57	(6.9)	1.80	(0.93-3.48)	0.081
	N/A	49	(44.1)	410	(49.9)			
	Missing	1	(0.9)	10	(1.2)			
Thrombotic event	*No*	111	(100)	801	(97.6)			
	Yes	0	(0)	7	(0.9)	0	(0.0-4.00)	0.325
	Missing	0	(0)	13	(1.6)			
Hypertensive disorder during pregnancy	*No*	77	(69.4)	631	(76.9)	1		
	Yes	33	(29.7)	183	(22.3)	1.48	(0.95-2.29)	0.082
	Missing	1	(0.9)	7	(0.9)			
Pregnancy induced hypertension	*No*	94	(84.7)	702	(85.5)	1		
	Yes	16	(14.4)	112	(13.6)	1.07	(0.61-1.88)	0.823

For peer review only

	Missing	1	(0.9)	7	(0.9)				
Pre-eclampsia/eclampsia	*No*	93	(83.8)	743	(90.5)	1			
	Yes	17	(15.3)	71	(8.6)	1.91		(1.08-3.39)	0.026
	Missing	1	(0.9)	7	(0.9)				

For peer review only

8 December 17

Table 3. Perinatal outcomes in women with BMI ≥ 60kg/m^2 and comparison women (BMI >50-<60kg/m^2).

		Number (%) of women BMI ≥ 60		Number (%) of women BMI>50-59		Unadjusted odds ratio	95% CI	P-Value
Perinatal death*	*No*	112	(98.2)	815	(98.5)	1		
	Yes	2	(1.8)	10	(1.2)	1.46	(0.31-6.74)	0.631
	Missing	0	(0)	2	(0.2)			
Still birth >24weeks gestation*	*No*	112	(98.2)	818	(98.9)	1		
	Yes	2	(1.8)	7	(0.8)	2.09	(0.43-10.19)	0.363
	Missing	0	(0)	2	(0.2)			
Preterm birth	*No*	101	(90.4)	730	(89)	1		
	Yes	10	(8.8)	87	(10.6)	0.83	(0.36-1.94)	0.668
	Missing	1	(0.9)	3	(0.4)			
Very preterm birth	*No*	111	(99.1)	804	(98)	Omitted		
	Yes	0	(0)	17	(1.6)			
	Missing	1	(0.9)	3	(0.4)			
Macrosomia (>4500grams)	*No*	98	(87.5)	746	(91.0)	1		
	Yes	14	(12.5)	72	(8.8)	1.48	(0.80-2.74)	0.211
	Missing	0	(0)	2	(0.2)			
Shoulder dystocia	*No*	44	(39.3)	373	(45.5)	1		
	Yes	1	(0.9)	19	(2.3)	0.45	(0.06-3.42)	0.438
	Missing	67	(59.8)	428	(52.5)			
Congenital abnormality	*No*	107	(95.5)	797	(97.2)	1		
	Yes	3	(2.7)	13	(1.6)	1.72	(0.48-6.14)	0.404
	Missing	2	(1.8)	10	(1.2)			
Infant respiratory problem	*No*	109	(97.3)	797	(97.2)	1		
	Yes	3	(2.7)	18	(2.2)	1.22	(0.35-4.21)	0.755
	Missing	0	(0)	5	(0.6)			
Apgar score <7 @ 5min	*No*	105	(93.8)	778	(94.9)	1		
	Yes	2	(1.8)	25	(3.0)	0.59	(0.14-2.54)	0.482
	Missing	5	(4.5)	17	(2.1)			

*Total birth denominator n=941. Odds ratios estimated using robust standard errors.

For peer review only

8 December 17

Supplementary table 1. Risk factors and administration of postnatal thromboprophylaxis in UK population: Green Top guideline no. 37a. Royal College of Obstetrics and Gynaecology

	Did not receive thromboprophylaxis		Received postnatal thromboprophylaxis	
Only risk factor BMI ≥40	7	(4.2)	3	(0.7)
Two or more risk factors	160	(95.8)	432	(99.3)
Should have received thromboprophylaxis**	**167**	**(27.7)**	435	(72.2)

*These included: caesarean section, age ≥35, infection, parity ≥3, smoker, preeclampsia, caesarean section, multiple births and stillbirth (Other risk factors were in the RCOG guideline that were not available in this dataset).

**Row percentage

Supplementary table 2. Administration for postnatal thromboprophylaxis and criteria for guideline in Australia

	Did not receive thromboprophylaxis		Received thromboprophylaxis	
Meet the guideline criteria	109	97.3	192	97.7
Did not meet the guideline criteria	3	2.7	4	2
Should have received thromboprophylaxis***	**109**	**(36.2)**	192	63.8

*Major risk factors included: caesarean section, preeclampsia, infection and BMI ≥35kg/m^2

**Minor risk factors included: Age > 35 years, smoker, post-partum haemorrhage and parity ≥3 (Other risk factors were in the guideline that were not available in this dataset).

***Row percentage

Summary for indication for post-partum prophylactic anticoagulation for South Australian Perinatal Practice Guidelines thromboprophylaxis and thromboembolic disease in pregnancy by South Australian Maternal & Neonatal Clinical Network

-Emergency caesarean section OR 2 or more major risk factors

-At least one major and 2 or more minor risk factors

For peer review only - http://bmjopen.bmj.com/site/about/guidelines.xhtml

STROBE 2007 (v4) checklist of items to be included in reports of observational studies in epidemiology*
Checklist for cohort, case-control, and cross-sectional studies (combined)

Section/Topic	Item #	Recommendation	Reported on page #
**Title and abstract**	1	(*a*) Indicate the study's design with a commonly used term in the title or the abstract	1
		(*b*) Provide in the abstract an informative and balanced summary of what was done and what was found	2
**Introduction**			
Background/rationale	2	Explain the scientific background and rationale for the investigation being reported	4
Objectives	3	State specific objectives, including any pre-specified hypotheses	5
**Methods**			
Study design	4	Present key elements of study design early in the paper	6
Setting	5	Describe the setting, locations, and relevant dates, including periods of recruitment, exposure, follow-up, and data collection	6
Participants	6	(*a*) *Cohort study*—Give the eligibility criteria, and the sources and methods of selection of participants. Describe methods of follow-up<br>*Case-control study*—Give the eligibility criteria, and the sources and methods of case ascertainment and control selection. Give the rationale for the choice of cases and controls<br>*Cross-sectional study*—Give the eligibility criteria, and the sources and methods of selection of participants	6
		(*b*) *Cohort study*—For matched studies, give matching criteria and number of exposed and unexposed<br>*Case-control study*—For matched studies, give matching criteria and the number of controls per case	
Variables	7	Clearly define all outcomes, exposures, predictors, potential confounders, and effect modifiers. Give diagnostic criteria, if applicable	6
Data sources/ measurement	8*	For each variable of interest, give sources of data and details of methods of assessment (measurement). Describe comparability of assessment methods if there is more than one group	*6-7 not reported in detail*
Bias	9	Describe any efforts to address potential sources of bias	6 not reported in detail
Study size	10	Explain how the study size was arrived at	7
Quantitative variables	11	Explain how quantitative variables were handled in the analyses. If applicable, describe which groupings were chosen and why	7-8
Statistical methods	12	(*a*) Describe all statistical methods, including those used to control for confounding	8
		(*b*) Describe any methods used to examine subgroups and interactions	8
		(*c*) Explain how missing data were addressed	7

For peer review only

		(*d*) *Cohort study*—If applicable, explain how loss to follow-up was addressed<br>*Case-control study*—If applicable, explain how matching of cases and controls was addressed<br>*Cross-sectional study*—If applicable, describe analytical methods taking account of sampling strategy	
		(*e*) Describe any sensitivity analyses	
**Results**			
Participants	13*	(a) Report numbers of individuals at each stage of study—eg numbers potentially eligible, examined for eligibility, confirmed eligible, included in the study, completing follow-up, and analysed	10
		(b) Give reasons for non-participation at each stage	N/A
		(c) Consider use of a flow diagram	N/A
Descriptive data	14*	(a) Give characteristics of study participants (eg demographic, clinical, social) and information on exposures and potential confounders	10
		(b) Indicate number of participants with missing data for each variable of interest	10-11, Tables
		(c) *Cohort study*—Summarise follow-up time (eg, average and total amount)	
Outcome data	15*	*Cohort study*—Report numbers of outcome events or summary measures over time	10-11, Tables
		Case-control study—Report numbers in each exposure category, or summary measures of exposure	
		Cross-sectional study—Report numbers of outcome events or summary measures	
Main results	16	(*a*) Give unadjusted estimates and, if applicable, confounder-adjusted estimates and their precision (eg, 95% confidence interval). Make clear which confounders were adjusted for and why they were included	10-11, Tables
		(*b*) Report category boundaries when continuous variables were categorized	
		(*c*) If relevant, consider translating estimates of relative risk into absolute risk for a meaningful time period	
Other analyses	17	Report other analyses done—eg analyses of subgroups and interactions, and sensitivity analyses	10-11
**Discussion**			
Key results	18	Summarise key results with reference to study objectives	12
Limitations	19	Discuss limitations of the study, taking into account sources of potential bias or imprecision. Discuss both direction and magnitude of any potential bias	12
Interpretation	20	Give a cautious overall interpretation of results considering objectives, limitations, multiplicity of analyses, results from similar studies, and other relevant evidence	12-14
Generalisability	21	Discuss the generalisability (external validity) of the study results	15
**Other information**			
Funding	22	Give the source of funding and the role of the funders for the present study and, if applicable, for the original study on which the present article is based	15

*Give information separately for cases and controls in case-control studies and, if applicable, for exposed and unexposed groups in cohort and cross-sectional studies.

For peer review only

Note: An Explanation and Elaboration article discusses each checklist item and gives methodological background and published examples of transparent reporting. The STROBE checklist is best used in conjunction with this article (freely available on the Web sites of PLoS Medicine at http://www.plosmedicine.org/, Annals of Internal Medicine at http://www.annals.org/, and Epidemiology at http://www.epidem.com/). Information on the STROBE Initiative is available at www.strobe-statement.org.

For peer review only

BMJ Open

A bi-national cohort study comparing the management and outcomes of pregnancy women with a BMI>50-59.9kg/m2 and those with a BMI 60kg/m2 or greater

Journal:	*BMJ Open*
Manuscript ID	bmjopen-2017-021055.R1
Article Type:	Research
Date Submitted by the Author:	02-Mar-2018
Complete List of Authors:	McCall, Stephen; University of Oxford Said Business School, National Perinatal Epidemiology Unit, Nuffield Department of Population Health<br>Li, Zhuoyang; University of Technology Sydney Faculty of Health, Australian Centre for Public and Population Health Research<br>Kurinczuk, Jenny; University of Oxford, National Perinatal Epidemiology Unit<br>Sullivan, Elizabeth; University of Technology Sydney Faculty of Health, Australian Centre for Public and Population Health Research<br>Knight, Marian; National Perinatal Epidemiology Unit
**Primary Subject Heading**:	Epidemiology
Secondary Subject Heading:	Obstetrics and gynaecology, Public health
Keywords:	Obesity, OBSTETRICS, obesity in pregnancy

SCHOLARONE™ Manuscripts

For peer review only

02/03/2018

A bi-national cohort study comparing the management and outcomes of pregnancy women with a BMI>50-59.9kg/m^2 and those with a BMI 60kg/m^2 or greater

Running title: The management, maternal and perinatal outcomes of women with a BMI 60kg/m^2 or greater

Stephen J. McCall[1]; Zhuoyang Li[2], Jennifer J. Kurinczuk[1], Elizabeth Sullivan[2]*, Marian Knight[1]*

[1] National Perinatal Epidemiology Unit (NPEU), Nuffield Department of Population Health, University of Oxford, Oxford, UK

[2]The Australian Centre for Public and Population Health Research, University of Technology Sydney, Sydney, Australia

*Joint senior authors

Address for correspondence: Stephen McCall, National Perinatal Epidemiology Unit, Nuffield Department of Population Health, University of Oxford, Richard Doll Building, Old Road Campus, Oxford, OX3 7LF, UK.

Email: Stephen.mccall@npeu.ox.ac.uk

Word count: 2960

For peer review only

02/03/2018

Abstract

Objectives: To compare the management, maternal and perinatal outcomes of women with a BMI 60kg/m^2 or greater with women with a BMI >50-59.9kg/m^2.

Design: International collaborative cohort study

Setting: Bi-national study in the UK and Australia

Participants: UK: all pregnant women and Australia: women gave birth (birthweight ≥400g or gestation ≥20 weeks)

Methods: Data from the Australasian Maternity Outcomes Surveillance System and UK Obstetric Surveillance System. Management, maternal and infant outcomes were compared between women with BMI 60kg/m^2 or greater and women with a BMI >50-59.9kg/m^2, using unconditional logistic regression.

Results: The sociodemographic characteristics and previous medical histories were similar between the 111 women with a BMI 60kg/m^2 or greater and the 821 women with a BMI >50-59.9kg/m^2. Women with a BMI 60kg/m^2 or greater had higher odds of thromboprophylaxis usage in both the antenatal (24% vs 12%; OR:2.25, 95%CI:1.39-3.64) and postpartum periods (78% vs 66%; OR:1.6, 95CI:1.04-2.70). Women with BMI 60kg/m^2 or greater had nearly double the odds of preeclampsia (adjusted OR:1.83 (95%CI:1.01-3.30)). No other maternal or perinatal outcomes were statistically significantly different. Severe adverse outcomes such as perinatal death were uncommon in both groups thus limiting the power of these comparisons. The rate of perinatal deaths was 18 per 1000 births for those with BMI 60kg/m^2 or greater ; 12.1 per 1000 births for those with BMI>50-59.9 kg/m^2; those with BMI 60kg/m^2 or greater had a non-significant increased odds of perinatal death (unadjusted OR:1.46, 95% CI:0.31-6.73).

For peer review only - http://bmjopen.bmj.com/site/about/guidelines.xhtml

Conclusions: Women are managed differently on the basis of BMI even at this extreme as shown by thromboprophylaxis. The preeclampsia result suggests that future research should examine whether weight reduction of any amount prior to pregnancy could reduce poor outcomes even if women remain extremely obese.

Strengths

- Population based study examining extreme obesity using national data from the UK and Australia.
- International collaborative studies allow the examination of rare exposures.

Limitations

-This study lacked the power to examine many maternal and perinatal outcomes despite having data from two national studies

-Some outcomes were not comparable between Australia and the UK so could not be explored.

For peer review only

02/03/2018

Introduction

Obesity is a major risk factor for non-communicable disease and morbidity in later life. It has reached epidemic levels in many high-income settings across all age-ranges. Obesity is defined as a body mass index (BMI) of ≥30 kg/m^2. Increasing rates of obesity in the general population are associated with an increasing trend towards obesity in pregnancy [1] Within the general population, the largest increases in obesity have been in the highest BMI groups [2] and this is also true for extreme obesity in pregnancy [3].

Maternal obesity is a risk factor for a number of pregnancy related complications and its relationship with these complications are complex [4, 5]. These relationships can be partially explained through pre-existing comorbidities such as diabetes [6], hypertension [6, 7] and asthma [6]. Pre-existing comorbidities have been shown to increase the risk of preeclampsia [8, 9] and venous thromboembolic events [10]. However, there remain other mechanisms that explain the association between obesity and preeclampsia/ venous thromboembolism, to specify a few, these are inflammation [11], insulin resistance [12] and oxidative stress [13, 14].

There have been several studies investigating the prevalence, outcomes and managements of extreme obesity in pregnancy (BMI ≥50 kg/m^2) [15-17]. These have aimed to test whether there was a dose response relationship between increased BMI and complications of pregnancy. Within the extremely obese group (BMI ≥50 kg/m^2) women included have had a BMI ranging from ≥50 kg/m^2 to approximately 75 kg/m^2. Whilst it may be the case that the risks rise exponentially with BMI it is possible that above a certain BMI, the risks of maternal and perinatal complications as a result of obesity do not increase due to the competing risks of other comorbidities. This remains to be investigated, as current published data do not allow the division of women into the highest BMI groups.

For peer review only - http://bmjopen.bmj.com/site/about/guidelines.xhtml

Previous research pooling together international data on rare exposures in pregnancy has been limited due to heterogeneity of definitions, methods and populations [18]. The obstetric surveillance systems in Australia and the UK were designed to be compatible with data collection using similar definitions with a view to pooling data. As a result, there are comparable data available to combine national studies, providing a large enough sample compare two groups of women within a cohort of extremely obese women. This study aimed to compare the characteristics, management including guideline adherence for prevention of venous thromboembolism, maternal and perinatal outcomes of women at the extremes of obesity.

For peer review only

Methods

Study population and design

This study was an international population-based cohort study, using secondary analysis of two national cohort studies of extreme maternal obesity, which were undertaken in Australia and the United Kingdom [16, 19]. For the purposes of the analysis, the exposed cohort were those pregnant women who had a BMI 60kg/m^2 or greater and the unexposed comparison cohort were those with a BMI >50-59.9 kg/m^2. Woman were included in the study if they had a BMI >50 kg/m^2 at any point during pregnancy and were included as part of the respective national studies [16, 19].

Anonymous data were prospectively collected using each of the national obstetric surveillance system the United Kingdom Obstetric Surveillance System (UKOSS) or Australasian Maternity Outcomes Surveillance System (AMOSS). The methods of each system have been described elsewhere in detail [19-21]. Briefly, in the UK, nominated reporters within each consultant-led obstetric unit received a monthly mailing card; the card had a tick box to indicate whether there had been a case of extreme obesity that month. There was also a box to indicate that there were no cases. Reporters returned cards regardless of whether there had been a case of extreme obesity. When a case was notified the reported received a data collection form. Using the medical records of the patient, information on demographic characteristics, obstetric history, medical history (including height and weight), management and outcomes were collected.

A similar method was used to identify women with extreme obesity in Australia. Designated reporters within each participating maternity unit within Australia were sent a monthly email. The reporter either responded with a “case” or a “nil case” to indicate whether there had truly been no cases. Once a case was reported, the reporter entered data on an online data

For peer review only - http://bmjopen.bmj.com/site/about/guidelines.xhtml

collection form using the case notes of the woman. The AMOSS system had 66% coverage of all women giving birth in Australia during the study [19].

Outcomes, management and potential covariates relevant to the research question were identified from the literature. On the basis of this, possible covariates and outcomes were identified in the respective UKOSS and AMOSS datasets. Each variable was mapped between the AMOSS and UKOSS datasets and an assessment of the comparability was made. On occasions, where the coding differed, harmonisation of the coding was devised and applied. This resulted in uniform values and labels of variables across both datasets. An assessment was made to determine whether the variables were measuring the same clinical phenotype in similar ways.

The covariates explored in the analysis were age, smoking status during pregnancy, previous pregnancy problems, pre-existing medical problems, pre-existing hypertension, parity and multiple pregnancies.

The missing data in similar datasets has been shown not to be missing at random; as a result multiple imputation was not considered appropriate [22]. A missing category was this created for each variable to account for the missing data. Primarily, complete case analysis was used in the multivariable analysis and a sensitivity analysis including the missing categories was used to assess the impact of missing data on the point estimates.

The sample size was predetermined by the size of the existing studies; therefore the sample was fixed at 111 women who had a BMI 60kg/m^2 or greater and 821 women who had a BMI >50-59.9kg/m^2. For the lowest frequency outcome (perinatal death), which had an incidence of 1.2% in the unexposed group, given the sample size the minimum odds ratio detectable as

statistically significant with 80% power at the 5% significance level was 5.63. For the highest frequency outcome, which had an incidence of 66.4% (thromboprophylaxis postnatally) in the unexposed group, the minimum odds ratio detectable as statistically significant with 80% power at the 5% significance level was 1.99.

Statistical analysis

Descriptive analyses were undertaken using the Chi Square test or Wilcoxon rank sum test as appropriate. These analyses assessed whether there was a statistical difference in characteristics between those women who had a BMI 60kg/m^2 or greater and those with a BMI >50-59 kg/m^2.

Each outcome was individually modelled in a univariable analysis using unconditional logistic regression, with results presented as unadjusted odds ratios (uOR) with 95% confidence intervals (95% CI). The exposure variable in each model was extreme obesity BMI 60kg/m^2 or greater. To account for clustering of infants within mothers' (multiple births) robust estimates of variance were calculated. Collinearity was assessed between all plausible linear associations prior to multivariable analysis, using Pearson's correlation coefficient.

Only outcomes that were statistically significant at the univariable level were included in the multivariable analysis. In the multivariable analysis, potential explanatory variables were sequentially added to the univariable model in a forward stepwise method with an examination of the results as each variable was added. A plausible explanatory variable was included in the final model if it was associated with the exposure and outcome (P-value for Wald test<0.05) or significantly improved the model fit assessed by likelihood ratio tests at the 5% significance level. Statistical analysis was completed using STATA V.13 (STATA CORP, Texas, USA).

For peer review only - http://bmjopen.bmj.com/site/about/guidelines.xhtml

A post hoc analysis was completed to assess the risk factors for venous thromboembolism possessed by those who did not receive postnatal thromboprophylaxis. This was a country specific analysis using risk factors of venous thromboembolism which were identified from the RCOG and South Australian Maternal & Neonatal Clinical Network guidelines [23, 24].

Patient and Public Involvement statement

There is PPI involvement in the UKOSS steering committee through lay members. The UKOSS steering committee assisted in the study design and management of the study. The AMOSS advisory group has PPI involvement through consumer, Maori and Pacific and Aboriginal and Torres Strait Islander members. The AMOSS advisory group provides advice on the implementation, delivery and development of the AMOSS system. The group also assists with the translation of findings into practice.

Ethics committee approval

The Australian collaborators obtained approval for the study from the NSW Population and Health Services Research Ethics Committee and multiple Human Research Ethics Committees across Australia [25]. Ethics committee approval for secondary analysis of anonymous UK data was not required.

For peer review only

Results

During the period September 2007-August 2008, 617 women with a BMI>50 kg/m^2 were identified through the UK Obstetric Surveillance System. Between January - October 2010, 315 women with a BMI>50 kg/m^2 were identified using the Australasian Maternity Outcomes Surveillance System. Overall there were 111 women with a BMI 60kg/m^2 or greater and 821 women with a BMI >50-59.9kg/m^2.

02/03/2018

Women with a BMI 60kg/m^2 or greater were slightly older, the sociodemographic characteristics and previous medical histories were otherwise similar the two groups of women (Table 1).

A high proportion in both groups experienced difficulties in visualisation of ultrasound (70.3% vs. 65.7%) although this was not statistically significant between the groups. Fewer women in both groups received antenatal thromboprophylaxis (24.3% BMI 60kg/m^2 or greater and 12.3% >50-59.9kg/m^2) compared to postnatal thromboprophylaxis (77.5% and 66.4%) (Table 2). Women with a BMI 60kg/m^2 or greater had a significantly higher odds of preeclampsia (uOR: 1.91 (95%CI: 1.08-3.39)), and of receiving either thromboprophylaxis antenatally (uOR:2.25 (95%CI:1.39-3.64)) or postnatally (uOR: 1.68 (95%CI: 1.04-2.70)) compared to those with a BMI >50-59.9kg/m^2 (Table 2). Supplementary tables 1 and 2 show that 27% and 32% of women should have received thromboprophylaxis postnatally in the UK and Australia as they had the relevant risk factors for it to be indicated , respectively.

Although not statistically significant, a higher proportion of women with a BMI 60kg/m^2 or greater experienced other adverse outcomes other than preeclampsia/eclampsia. Preeclampsia/eclampsia was examined in a multivariable model. The presence of a BMI 60kg/m^2 or greater was associated with a two-fold increase in the odds of having preeclampsia/eclampsia (aOR:1.83 (95%CI: 1.01-3.30)) compared to those with a BMI >50-59kg/m^2, after adjusting for smoking status, pre-existing diabetes and parity. The results of the proxy variable model did not materially differ to those of the complete case analysis.

Severe adverse outcomes such as perinatal death were uncommon in both groups (n=2 (18 per 1000 births), BMI 60kg/m^2 or greater vs. n=10 (12 per 1000 births), BMI $\geq$ 50-59kg/m^2). There were no statistically significant differences in perinatal outcomes between both obesity groups (see table 3).

For peer review only - http://bmjopen.bmj.com/site/about/guidelines.xhtml

02/03/2018

For peer review only

02/03/2018

Discussion

Main Findings

Compared with women with a BMI >50-59 women with a BMI 60kg/m^2 or greater had an increased risk of preeclampsia/eclampsia, There were very few statistically significant differences in outcomes between the two very high BMI groups. Nevertheless, the direction of effects favours the lower BMI group for most outcomes. Further research should test whether any weight reduction could reduce poor outcomes even if women remain extremely obese. Importantly, the perinatal mortality rate was higher in both groups compared with both the UK/Australian rate. Women are being managed differently on the basis of BMI even at this extreme as use of thromboprophylactic drugs varied between the two high BMI groups.

Strengths and limitations

Both prospective population based surveillance systems use a robust methodology, which reduces the risk of selection bias. Two national studies allowed the examination of women with a BMI 60kg/m^2 or greater in a high resource setting and thus overcomes some of the limitations of previous research which was limited by the number of women in the extreme ends of the BMI distribution. Nevertheless, despite pooling of national data the number of women in each group was still relatively small, which limited the study power, particularly when investigating rare outcomes.

This study did not have access to ethnicity from Australia and socioeconomic measures were not comparable between the countries. Thus the adjusted odd ratio presented may be vulnerable to residual confounding if ethnicity and socioeconomic status were associated with both the outcome and exposure.

For peer review only - http://bmjopen.bmj.com/site/about/guidelines.xhtml

Interpretation

One of the novel benefits of this multi-national study was the ability to examine a subset of the more extreme end of the spectrum of obesity. This demonstrated that women with a BMI 60kg/m^2 or greater had very similar characteristics and experienced similar management compared to women with a BMI>50 -<60 kg/m^2. Interestingly, the BMI 60kg/m^2 or greater group had an increased risk of preeclampsia/eclampsia; which supports the hypothesis of a 'dose response' relationship between obesity and preeclampsia/eclampsia seen at lower BMIs [26] and super obesity [17, 27].

The comparison of extreme maternal obesity and a representative BMI group has been previously studied [16, 19]. The risk of preeclampsia, venous thromboembolism, preterm delivery, shoulder dystocia, caesarean delivery was elevated in women with extreme maternal obesity compared to non-extremely obese women [16, 19]. Despite few statistically significant differences in outcomes between the two groups, the literature highlights that the risk is substantially higher for extremely obese women compared to women in a normal BMI group.

The BMI 60kg/m^2 or greater cohort had a higher proportion of perinatal deaths and stillbirths than the BMI >50-59.9 kg/m^2 cohort, although these were not statistically significantly different possibly because of the small numbers involved. The absolute rate of perinatal death for the 60kg/m^2 or greater cohort was three times higher than the UK rate (5.6 per 1000 births) and 2.5 times higher than the Australian rate (7.3 per 1000); while the rate of perinatal mortality in the >50-59.9 kg/m^2 cohort was just over twice that of the UK rate and was 1.5 times higher than the Australian perinatal mortality rate [28, 29].

A previous study of extreme obesity that examine perinatal outcomes has suggested that there is a dose response relationship between BMI and perinatal outcomes [17]. The small sample

For peer review only - http://bmjopen.bmj.com/site/about/guidelines.xhtml

size and relative rarity of adverse perinatal outcomes in this analysis did not allow the role of chance to be excluded for most outcomes even with pooling two national studies.

The results of this study show that the degree of relative obesity impacted on thromboprophylaxis practice. The Royal College of Obstetricians and Gynaecologists' guideline states that any women with a BMI >40kg/m^2 should be considered at intermediate risk of venous thromboembolism and should be given at least 10 days of thromboprophylaxis postnatally [23]. Within Australia there is regional variation in the guidelines concerning BMI and postpartum thromboprophylaxis. The Queensland state guideline suggests that a woman must possess three or more risk factors (BMI >30 kg/m^2 being one of these risk factors) to be given low molecular weight heparin for 6 days postnatally, while the South Australian government and current expert opinion recommends that BMI ≥30 kg/m^2 plus one major risk factor for thromboembolism requires prophylactic anticoagulation for 5 days postpartum [24, 30]. Data from this study suggests guidelines appear to be followed variably due to the large variation in practice between the two cohorts. This suggests more implementation work within clinical settings is needed to help these guidelines be followed. Nevertheless, the results show that BMI has an important impact on clinical decisions concerning the administration of thromboprophylaxis postnatally.

Importantly, approximately 75% had postnatal thromboprophylaxis which is smaller than expected considering this was an extremely obese population. Nearly a third of women in both countries had the appropriate risk factors to indicate the use of thromboprophylaxis postnatally. This highlights an important area for improvement of clinical practice to prevent a potentially fatal venous thromboembolism.

Interestingly, there were no venous thromboembolic events in the BMI 60kg/m^2 or greater group, which was the group in which the larger proportion of women received

For peer review only - http://bmjopen.bmj.com/site/about/guidelines.xhtml

thromboprophylaxis although again these are very rare events. Previous studies have shown that BMI is a strong risk factor of venous thromboembolism [31, 32] and the risk is amplified in those who have a high BMI and were immobilized [33].

This study was a secondary data analysis of women identified during 2008 in the UK and 2010 in the Australia. As a result, it is likely that the proportion of women who have a BMI >50 since the original studies is likely to be much larger which makes the findings of this study even more pertinent.

Conclusions

There were very few statistically significant differences in outcomes between these two high BMI groups. However, the direction of effect favours the lower BMI group for most outcomes and a type II error cannot be excluded given the small number of outcomes. Preeclampsia risk is increased with increasing BMI in the morbidly obese women. Further research should test whether any weight reduction could reduce poor outcomes even if women remain extremely obese. Women are clearly being managed differently on the basis of BMI even at this extreme as shown by the thromboprophylaxis data. Furthermore, there was a failure to full apply thromboprophylaxis guidelines fully in 2007-2008 which emphasises a need to ensure women at risk of venous thromboembolism receive appropriate prevention care.

Acknowledgements: We thank the AMOSS advisory group and UKOSS steering committee for their input into the respective obesity studies. Dr Manisha Nair and Dr Bryn Kemp for their advice on the project.

Data sharing statement: The NPEU Data sharing agreement can be found here: https://www.npeu.ox.ac.uk/downloads/files/npeu/policies/Data%20Sharing%20Policy.pdf

For peer review only

Access to the Australian data must be made to the AMOSS steering committee.

Disclosures of interest: None

Funding: SM is funded by the Nuffield Department of Population Health and Medical Research Council (MRC) training grant MR/K501256/1. Marian Knight is funded by an NIHR Research Professorship. The views expressed in this publication are those of the author(s), and not necessarily those of the MRC, the NHIR or the Department of Health. The funder had no role in the study design, data collection and analysis, decision to publish, or preparation of the article. The National Health and Medical Research Council Project Grant (Application 510298) for funding The Australian Maternity Outcomes Surveillance System: Improving safety and quality of maternity care in Australia (AMOSS) from 2008–2012.

Author contribution: MK, JK, ES conceived the study. MK, JK, ES and SM designed the study. ZL extracted the Australian data. SM analysed the data and wrote the first draft. All authors interpreted the data and edited the manuscript.

Tables list

Table 1. Sociodemographic characteristics and previous medical problems in women with BMI 60kg/m^2 or greater and comparison women (BMI >50-59.9kg/m^2).

Table 2. Maternal outcomes and management in women with BMI 60kg/m^2 or greater and comparison women (BMI >50-59.9kg/m^2).

Table 3. Perinatal outcomes in women with BMI 60kg/m^2 or greater and comparison women (BMI >50-59.9kg/m^2).

For peer review only

For peer review only - http://bmjopen.bmj.com/site/about/guidelines.xhtml

References

1. HESLEHURST, N., RANKIN, J., WILKINSON, J. R. & SUMMERBELL, C. D. 2010. A nationally representative study of maternal obesity in England, UK: Trends in incidence and demographic inequalities in 619 323 births, 1989-2007. *International Journal of Obesity,* 34**,** 420-428.
2. STURM, R. 2007. Increases in morbid obesity in the USA: 2000–2005. *Public Health,* 121**,** 492-496.
3. KIM, S. Y., DIETZ, P. M., ENGLAND, L., MORROW, B. & CALLAGHAN, W. M. 2007. Trends in Pre□pregnancy Obesity in Nine States, 1993–2003. *Obesity,* 15**,** 986-993.
4. BHATTACHARYA, S., CAMPBELL, D. M., LISTON, W. A. & BHATTACHARYA, S. 2007. Effect of body mass index on pregnancy outcomes in nulliparous women delivering singleton babies. *BMC public Health,* 7**,** 168.
5. CATALANO, P. M. & EHRENBERG, H. M. 2006. Review article: The short- and long-term implications of maternal obesity on the mother and her offspring. *BJOG: An International Journal of Obstetrics & Gynaecology,* 113**,** 1126-1133.
6. MOKDAD, A.H., FORD, E.S., BOWMAN, B.A., DIETZ, W.H., VINICOR, F., BALES, V.S. and MARKS, J.S., 2003. Prevalence of obesity, diabetes, and obesity-related health risk factors, 2001. *JAMA,* 289(1), pp.76-79.
7. RE, R.N., 2009. Obesity-related hypertension. *The Ochsner Journal,* 9(3), pp.133-136.
8. DUCKITT, K. and HARRINGTON, D., 2005. Risk factors for pre-eclampsia at antenatal booking: systematic review of controlled studies. *BMJ,* 330(7491), p.565.
9. SIBIA, B.M., Gordon, T., Thom, E., Caritis, S.N., Klebanoff, M., McNellis, D. and Paul, R.H., 1995. Risk factors for preeclampsia in healthy nulliparous women: a prospective multicenter study. *American Journal of Obstetrics & Gynecology,* 172(2), pp.642-648.
10. JAMES, A.H., JAMISON, M.G., BRANCAZIO, L.R. and MYERS, E.R., 2006. Venous thromboembolism during pregnancy and the postpartum period: incidence, risk factors, and mortality. *American Journal of Obstetrics & Gynecology,* 194(5), pp.1311-13.
11. WOLF, M., KETTYLE, E., SANDLER, L., ECKER, J. L., ROBERTS, J. & THADHANI, R. 2001. Obesity and preeclampsia: the potential role of inflammation. *Obstetrics & Gynecology,* 98**,** 757-762.
12. VAN GAAL, L. F., MERTENS, I. L. & CHRISTOPHE, E. 2006. Mechanisms linking obesity with cardiovascular disease. *Nature,* 444**,** 875.
13. ROBERTS, J. M., BODNAR, L. M., PATRICK, T. E. & POWERS, R. W. 2011. The role of obesity in preeclampsia. *Pregnancy Hypertension: An International Journal of Women's Cardiovascular Health,* 1**,** 6-16.
14. FREEDMAN, J. E. 2008. Oxidative stress and platelets. *Arteriosclerosis, thrombosis, and vascular biology,* 28**,** s11-s16.
15. MARTIN, A., KRISHNA, I., ELLIS, J., PACCIONE, R. & BADELL, M. 2014. Super obesity in pregnancy: difficulties in clinical management. *Journal of Perinatology,* 34**,** 495-502.
16. KNIGHT, M., KURINCZUK, J. J., SPARK, P., BROCKLEHURST, P. & SYSTEM, U. K. O. S. 2010. Extreme obesity in pregnancy in the United Kingdom. *Obstetrics & Gynecology,* 115**,** 989-97.

For peer review only

17. MARSHALL, N. E., GUILD, C., CHENG, Y. W., CAUGHEY, A. B. & HALLORAN, D. R. 2012. Maternal superobesity and perinatal outcomes. *American Journal of Obstetrics & Gynecology,* 206**,** 417.e1-6.
18. KNIGHT, M., CALLAGHAN, W. M., BERG, C., ALEXANDER, S., BOUVIER-COLLE, M. H., FORD, J. B., JOSEPH, K. S., LEWIS, G., LISTON, R. M., ROBERTS, C. L., OATS, J. & WALKER, J. 2009. Trends in postpartum hemorrhage in high resource countries: a review and recommendations from the International Postpartum Hemorrhage Collaborative Group. *BMC Pregnancy & Childbirth,* 9**,** 55.
19. SULLIVAN, E. A., DICKINSON, J. E., VAUGHAN, G. A., PEEK, M. J., ELLWOOD, D., HOMER, C. S. E., KNIGHT, M., MCLINTOCK, C., WANG, A., POLLOCK, W., JACKSON PULVER, L., LI, Z., JAVID, N., DENNEY-WILSON, E. & CALLAWAY, L. 2015. Maternal super-obesity and perinatal outcomes in Australia: A national population-based cohort study. *BMC Pregnancy and Childbirth,* 15:322
20. KNIGHT, M., KURINCZUK, J. J., TUFFNELL, D. & BROCKLEHURST, P. 2005. The UK Obstetric Surveillance System for rare disorders of pregnancy. *BJOG: An International Journal of Obstetrics and Gynaecology,* 112**,** 263-265.
21. MCDONNELL, N., KNIGHT, M., PEEK, M. J., ELLWOOD, D., HOMER, C. S. E., MCLINTOCK, C., VAUGHAN, G., POLLOCK, W., LI, Z., JAVID, N. & SULLIVAN, E. 2015. Amniotic fluid embolism: an Australian-New Zealand population-based study. *BMC Pregnancy and Childbirth,* 15**,** 352.
22. LINDQUIST, A., KNIGHT, M. & KURINCZUK, J. J. 2013. Variation in severe maternal morbidity according to socioeconomic position: a UK national case–control study. *BMJ open,* 3**,** e002742.
23. ROYAL COLLEGE OF OBSTETRICS AND GYNAECOLOGY (RCOG) 2015. Reducing the Risk of Venous Thromboembolism during Pregnancy and the Puerperium. Green-top Guideline No. 37a. London: RCOG.
24. SOUTH AUSTRALIAN MATERNAL & NEONATAL CLINICAL NETWORK. 2013. South Australian Perinatal Practice Guidelines: thromboprophylaxis and thromboembolic disease in pregnancy. Department of Health, Government of South Australia.
25. VAUGHAN, G., POLLOCK, W., PEEK, M. J., KNIGHT, M., ELLWOOD, D., HOMER, C. S., PULVER, L. J., MCLINTOCK, C., HO, M. T. & SULLIVAN, E. A. 2012. Ethical issues: The multi-centre low-risk ethics/governance review process and AMOSS. *Australian and New Zealand Journal of Obstetrics and Gynaecology,* 52**,** 195-203.
26. BODNAR, L. M., CATOV, J. M., KLEBANOFF, M. A., NESS, R. B. & ROBERTS, J. M. 2007. Prepregnancy body mass index and the occurrence of severe hypertensive disorders of pregnancy. *Epidemiology,* 18**,** 234-239.
27. MBAH, A. K., KORNOSKY, J. L., KRISTENSEN, S., AUGUST, E. M., ALIO, A. P., MARTY, P. J., BELOGOLOVKIN, V., BRUDER, K. & SALIHU, H. M. 2010. Super-obesity and risk for early and late pre-eclampsia. *BJOG: An International Journal of Obstetrics and Gynaecology,* 117**,** 997-1003.
28. MANKTELOW, B., SMITH, L., PRUNET, C., SMITH, P., BOBY, T., HYMAN-TAYLOR, P., KURINCZUK, J., FIELD, D., E., D. & ON BEHALF OF THE MBRRACE-UK COLLABORATION 2017. *MBRRACE-UK Perinatal Mortality Surveillance Report: UK Perinatal Deaths for Births from January to December 2015*, Department of Health Sciences, University of Leicester.
29. MONK, A., HARRIS, K., DONNOLLEY, N., HILDER, L., HUMPHREY, M., GORDON, A. & CHAMBERS, G on behalf of Austrailian Institute of Health and

For peer review only

02/03/2018

Welfare.. 2016. Perinatal deaths in Australia, 1993–2012. Perinatal deaths. Canberra: Austrailian Institute of Health and Welfare.

30. MCLINTOCK, C., BRIGHTON, T., CHUNILAL, S., DEKKER, G., MCDONNELL, N., MCRAE, S., MULLER, P., TRAN, H., WALTERS, B. N. & YOUNG, L. 2012. Recommendations for the prevention of pregnancy□associated venous thromboembolism. *Australian and New Zealand Journal of Obstetrics and Gynaecology,* 52**,** 3-13.
31. LARSEN, T. B., SØRENSEN, H. T., GISLUM, M. & JOHNSEN, S. P. 2007. Maternal smoking, obesity, and risk of venous thromboembolism during pregnancy and the puerperium: A population-based nested case-control study. *Thrombosis Research,* 120**,** 505-509.
32. KNIGHT, M. 2008. Antenatal pulmonary embolism: risk factors, management and outcomes. *BJOG: An International Journal of Obstetrics & Gynaecology,* 115**,** 453-461.
33. JACOBSEN, A. F., SKJELDESTAD, F. E. & SANDSET, P. M. 2008. Ante- and postnatal risk factors of venous thrombosis: a hospital-based case–control study. *Journal of Thrombosis and Haemostasis,* 6**,** 905-912.

For peer review only

For peer review only - http://bmjopen.bmj.com/site/about/guidelines.xhtml

02/03/2018

Table 1. Sociodemographic characteristics and previous medical problems in women with BMI 60kg/m^2 or greater and comparison women (BMI >50-59.9kg/m^2).

**Characteristic**		**Number (%) of obese women BMI 60 or greater (n=111)**		**Number (%) of women BMI 50 - 59.9 (n=821)**		**p-value**
Sociodemographic characteristics						
Age	*Mean (Std)*	31.7	(5.51)	30.3	(5.67)	0.017
BMI at booking	*Median (IQR)*	61.7	(60-64.9)	52.3	(50.8-54.9)	<0.001
Max recorded BMI	*Median (IQR)*	62.9	(61-66.8)	52.7	(50.9-55.0)	<0.001
Smoking status	*Never/ex-smoker*	85	(76.6)	599	(73)	0.42
	Smoked during pregnancy	24	(21.6)	206	(25.1)	
	Missing	2	(1.8)	16	(1.9)	
Known previous medical history						
Previous pregnancy problems	*None*	41	(36.9)	273	(33.3)	0.713
	Yes	33	(29.7)	266	(32.4)	
	Not applicable	35	(31.5)	271	(33.0)	
	Missing	2	(1.8)	11	(1.3)	
Known cardiac disease	*None*	109	(98.2)	812	(98.9)	0.174
	Yes	2	(1.8)	5	(0.6)	
	Missing	0	(0)	4	(0.5)	
Known renal disease	*None*	110	(99.1)	809	(98.5)	0.937
	Yes	1	(0.9)	8	(1.0)	
	Missing	0	(0)	4	(0.5)	
Known mental health issues	*None*	99	(89.2)	756	(92.1)	0.219
	Yes	12	(10.8)	61	(7.4)	
	Missing	0	(0)	4	(0.5)	
Known asthma	*None*	98	(88.3)	720	(87.7)	0.961
	Yes	13	(11.7)	97	(11.8)	
	Missing	0	(0)	4	(0.5)	
Previous caesarean delivery	*None*	53	(47.7)	366	(44.6)	0.297
	Yes	22	(19.8)	181	(22.0)	
	Not applicable	35	(31.5)	271	(33.0)	
	Missing	1	(0.9)	3	(0.4)	
Parity	*Nulliparous*	35	(31.5)	271	(33)	0.803
	Multiparous	75	(67.6)	550	(67)	
	Missing	1	(0.9)	0	(0)	
Current pregnancy						

For peer review only

For peer review only - http://bmjopen.bmj.com/site/about/guidelines.xhtml

02/03/2018

Multiple pregnancy	*Singleton*	108	(97.3)	800	(97.4)	0.928
	Twin pregnancy	3	(2.7)	21	(2.6)	
Known hypertension prior to pregnancy requiring treatment	*None*	103	(92.8)	767	(93.4)	0.693
	Yes	8	(7.2)	51	(6.2)	
	Missing	0	(0)	3	(0.4)	
Known pre-existing diabetes prior to pregnancy	*None*	101	(91.0)	757	(92.2)	0.657
	Yes	10	(9.0)	64	(7.8)	
	Missing	-	-	-	-	
Insulin dependent diabetes	*Yes*	4	(3.6)	17	(2.1)	0.663

For peer review only

02/03/2018

Table 2. Maternal outcomes and management in women with BMI 60kg/m^2 or greater and comparison women (BMI >50-59.9kg/m^2).

		Number (%) of women BMI 60 or greater (n=111)		Number (%) of women BMI >50-59.9(n=821)		Unadjusted odds ratio	95% Confidence interval	P-value
Management								
Difficulties undertaking	*No*	30	(27)	228	(27.8)	1		
ultrasounds	*Yes*	78	(70.3)	539	(65.7)	1.10	(0.70-1.72)	0.678
	Missing	3	(2.7)	54	(6.6)			
Induced	*No*	59	(53.2)	405	(49.3)	1		
	Yes	40	(36)	303	(36.9)	0.91	(0.59-1.39)	0.652
	Missing	12	(10.8)	113	(13.8)			
Syntocinon	*No*	25	(22.5)	243	(29.6)	1		
	Yes	41	(36.9)	289	(35.2)	1.38	(0.82-2.33)	0.231
	Missing	45	(40.5)	289	(35.2)			
Caesarean delivery	*No*	48	(43.2)	398	(48.5)	1		.
	Yes	62	(55.9)	411	(50.1)	1.25	(0.84,1.87)	0.274
	Missing	1	(0.9)	12	(1.5)			
Thromboprophylaxis usage antenatal	*No*	84	(75.7)	706	(86)	1		
	Yes	27	(24.3)	101	(12.3)	2.25	(1.39-3.64)	0.001
	Missing	0	(0)	14	(1.7)			
Thromboprophylaxis post	*No*	24	(21.6)	255	(31.1)	1		
	Yes	86	(77.5)	545	(66.4)	1.68	(1.04-2.70)	0.033
	Missing	1	(0.9)	21	(2.6)			
Maternal Outcome								
Wound infection in those with caesarean	*No*	47	(42.3)	344	(41.9)	1		
	Yes	14	(12.6)	57	(6.9)	1.80	(0.93-3.48)	0.081
	N/A	49	(44.1)	410	(49.9)			
	Missing	1	(0.9)	10	(1.2)			
Venous thromboembolism *	*No*	111	(100)	807	(98.3)			
	Yes	0	(0)	7	(0.9)	0	(0.0-4.00)	0.325
	Missing	0	(0)	7	(0.9)			
Hypertensive disorder during pregnancy	*No*	77	(69.4)	631	(76.9)	1		
	Yes	33	(29.7)	183	(22.3)	1.48	(0.95-2.29)	0.082
	Missing	1	(0.9)	7	(0.9)			
Pregnancy induced hypertension	*No*	94	(84.7)	702	(85.5)	1		
	Yes	16	(14.4)	112	(13.6)	1.07	(0.61-1.88)	0.823

For peer review only

For peer review only - http://bmjopen.bmj.com/site/about/guidelines.xhtml

	Missing	1	(0.9)	7	(0.9)				
Pre-eclampsia/eclampsia	*No*	93	(83.8)	743	(90.5)	1			
	Yes	17	(15.3)	71	(8.6)	1.91		(1.08-3.39)	0.026
	Missing	1	(0.9)	7	(0.9)				

For peer review only

For peer review only - http://bmjopen.bmj.com/site/about/guidelines.xhtml

2 March 18~~2 March 2018~~

Table 3. Perinatal outcomes in women with BMI 60kg/m^2 or greater and comparison women (BMI >50-59.9kg/m^2).

		**Number (%) of women BMI 60 or greater**		**Number (%) of women BMI >50-59.9**		**Unadjusted odds ratio**	**95% CI**	**P-Value**
Perinatal death*	*No*	112	(98.2)	815	(98.5)	1		
	Yes	2	(1.8)	10	(1.2)	1.46	(0.31-6.74)	0.631
	Missing	0	(0)	2	(0.2)			
Still birth >24weeks gestation*	*No*	112	(98.2)	818	(98.9)	1		
	Yes	2	(1.8)	7	(0.8)	2.09	(0.43-10.19)	0.363
	Missing	0	(0)	2	(0.2)			
Preterm birth	*No*	101	(90.2)	730	(89)	1		
	Yes	10	(8.9)	87	(10.6)	0.83	(0.36-1.94)	0.668
	Missing	1	(0.9)	3	(0.4)			
Very preterm birth	*No*	111	(99.1)	804	(98)		Omitted	
	Yes	0	(0)	17	(1.6)			
	Missing	1	(0.9)	3	(0.4)			
Birthweight	*Mean (Std)*	3683.0	(752.1)	3603.7	(715.0)		Omitted	
Macrosomia (>4500grams)	*No*	98	(87.5)	746	(91.0)	1		
	Yes	14	(12.5)	72	(8.8)	1.48	(0.80-2.74)	0.211
	Missing	0	(0)	2	(0.2)			
Shoulder dystocia	*No*	44	(39.3)	373	(45.5)	1		
	Yes	1	(0.9)	19	(2.3)	0.45	(0.06-3.42)	0.438
	Missing	67	(59.8)	428	(52.5)			
Congenital abnormality	*No*	107	(95.5)	797	(97.2)	1		
	Yes	3	(2.7)	13	(1.6)	1.72	(0.48-6.14)	0.404
	Missing	2	(1.8)	10	(1.2)			
Infant respiratory problem	*No*	109	(97.3)	797	(97.2)	1		
	Yes	3	(2.7)	18	(2.2)	1.22	(0.35-4.21)	0.755
	Missing	0	(0)	5	(0.6)			
Apgar score <7 @ 5min	*No*	105	(93.8)	778	(94.9)	1		
	Yes	2	(1.8)	25	(3.0)	0.59	(0.14-2.54)	0.482
	Missing	5	(4.5)	17	(2.1)			

Odds ratios estimated using robust standard errors. *Denominator is birth (including multiple births) and stillbirths n=941. Denominator in the remainder of the table is live births (including multiple births).

For peer review only

For peer review only - http://bmjopen.bmj.com/site/about/guidelines.xhtml

For peer review only

8 December 17

Supplementary table 1. Risk factors and administration of postnatal thromboprophylaxis in UK population: Green Top guideline no. 37a. Royal College of Obstetrics and Gynaecology

	Did not receive thromboprophylaxis		Received postnatal thromboprophylaxis	
Only risk factor BMI ≥40	7	(4.2)	3	(0.7)
Two or more risk factors	160	(95.8)	432	(99.3)
Should have received thromboprophylaxis**	**167**	**(27.7)**	435	(72.2)

*These included: caesarean section, age ≥35, infection, parity ≥3, smoker, preeclampsia, caesarean section, multiple births and stillbirth (Other risk factors were in the RCOG guideline that were not available in this dataset).

**Row percentage

Supplementary table 2. Administration for postnatal thromboprophylaxis and criteria for guideline in Australia

	Did not receive thromboprophylaxis		Received thromboprophylaxis	
Meet the guideline criteria	109	97.3	192	97.7
Did not meet the guideline criteria	3	2.7	4	2
Should have received thromboprophylaxis***	**109**	**(36.2)**	192	63.8

*Major risk factors included: caesarean section, preeclampsia, infection and BMI ≥35kg/m^2

**Minor risk factors included: Age > 35 years, smoker, post-partum haemorrhage and parity ≥3 (Other risk factors were in the guideline that were not available in this dataset).

***Row percentage

Summary for indication for post-partum prophylactic anticoagulation for South Australian Perinatal Practice Guidelines thromboprophylaxis and thromboembolic disease in pregnancy by South Australian Maternal & Neonatal Clinical Network

-Emergency caesarean section OR 2 or more major risk factors

-At least one major and 2 or more minor risk factors

For peer review only - http://bmjopen.bmj.com/site/about/guidelines.xhtml

STROBE 2007 (v4) checklist of items to be included in reports of observational studies in epidemiology*

Checklist for cohort, case-control, and cross-sectional studies (combined)

Section/Topic	Item #	Recommendation	Reported on page #
**Title and abstract**	1	(*a*) Indicate the study's design with a commonly used term in the title or the abstract	1
		(*b*) Provide in the abstract an informative and balanced summary of what was done and what was found	2
**Introduction**			
Background/rationale	2	Explain the scientific background and rationale for the investigation being reported	4
Objectives	3	State specific objectives, including any pre-specified hypotheses	5
**Methods**			
Study design	4	Present key elements of study design early in the paper	6
Setting	5	Describe the setting, locations, and relevant dates, including periods of recruitment, exposure, follow-up, and data collection	6
Participants	6	(*a*) *Cohort study*—Give the eligibility criteria, and the sources and methods of selection of participants. Describe methods of follow-up<br>*Case-control study*—Give the eligibility criteria, and the sources and methods of case ascertainment and control selection. Give the rationale for the choice of cases and controls<br>*Cross-sectional study*—Give the eligibility criteria, and the sources and methods of selection of participants	6
		(*b*) *Cohort study*—For matched studies, give matching criteria and number of exposed and unexposed<br>*Case-control study*—For matched studies, give matching criteria and the number of controls per case	
Variables	7	Clearly define all outcomes, exposures, predictors, potential confounders, and effect modifiers. Give diagnostic criteria, if applicable	6
Data sources/ measurement	8*	For each variable of interest, give sources of data and details of methods of assessment (measurement). Describe comparability of assessment methods if there is more than one group	*6-7 not reported in detail*
Bias	9	Describe any efforts to address potential sources of bias	6 not reported in detail
Study size	10	Explain how the study size was arrived at	7
Quantitative variables	11	Explain how quantitative variables were handled in the analyses. If applicable, describe which groupings were chosen and why	7-8
Statistical methods	12	(*a*) Describe all statistical methods, including those used to control for confounding	8
		(*b*) Describe any methods used to examine subgroups and interactions	8
		(*c*) Explain how missing data were addressed	7

For peer review only

		(*d*) *Cohort study*—If applicable, explain how loss to follow-up was addressed<br>*Case-control study*—If applicable, explain how matching of cases and controls was addressed<br>*Cross-sectional study*—If applicable, describe analytical methods taking account of sampling strategy	
		(*e*) Describe any sensitivity analyses	
**Results**			
Participants	13*	(a) Report numbers of individuals at each stage of study—eg numbers potentially eligible, examined for eligibility, confirmed eligible, included in the study, completing follow-up, and analysed	10
		(b) Give reasons for non-participation at each stage	N/A
		(c) Consider use of a flow diagram	N/A
Descriptive data	14*	(a) Give characteristics of study participants (eg demographic, clinical, social) and information on exposures and potential confounders	10
		(b) Indicate number of participants with missing data for each variable of interest	10-11, Tables
		(c) *Cohort study*—Summarise follow-up time (eg, average and total amount)	
Outcome data	15*	*Cohort study*—Report numbers of outcome events or summary measures over time	10-11, Tables
		Case-control study—Report numbers in each exposure category, or summary measures of exposure	
		Cross-sectional study—Report numbers of outcome events or summary measures	
Main results	16	(*a*) Give unadjusted estimates and, if applicable, confounder-adjusted estimates and their precision (eg, 95% confidence interval). Make clear which confounders were adjusted for and why they were included	10-11, Tables
		(*b*) Report category boundaries when continuous variables were categorized	
		(*c*) If relevant, consider translating estimates of relative risk into absolute risk for a meaningful time period	
Other analyses	17	Report other analyses done—eg analyses of subgroups and interactions, and sensitivity analyses	10-11
**Discussion**			
Key results	18	Summarise key results with reference to study objectives	12
Limitations	19	Discuss limitations of the study, taking into account sources of potential bias or imprecision. Discuss both direction and magnitude of any potential bias	12
Interpretation	20	Give a cautious overall interpretation of results considering objectives, limitations, multiplicity of analyses, results from similar studies, and other relevant evidence	12-14
Generalisability	21	Discuss the generalisability (external validity) of the study results	15
**Other information**			
Funding	22	Give the source of funding and the role of the funders for the present study and, if applicable, for the original study on which the present article is based	15

*Give information separately for cases and controls in case-control studies and, if applicable, for exposed and unexposed groups in cohort and cross-sectional studies.

For peer review only

Note: An Explanation and Elaboration article discusses each checklist item and gives methodological background and published examples of transparent reporting. The STROBE checklist is best used in conjunction with this article (freely available on the Web sites of PLoS Medicine at http://www.plosmedicine.org/, Annals of Internal Medicine at http://www.annals.org/, and Epidemiology at http://www.epidem.com/). Information on the STROBE Initiative is available at www.strobe-statement.org.

For peer review only

BMJ Open

A bi-national cohort study comparing the management and outcomes of pregnancy women with a BMI>50-59.9kg/m2 and those with a BMI 60kg/m2 or greater

Journal:	*BMJ Open*
Manuscript ID	bmjopen-2017-021055.R2
Article Type:	Research
Date Submitted by the Author:	05-Jun-2018
Complete List of Authors:	McCall, Stephen; University of Oxford Said Business School, National Perinatal Epidemiology Unit, Nuffield Department of Population Health<br>Li, Zhuoyang; University of Technology Sydney Faculty of Health, Australian Centre for Public and Population Health Research<br>Kurinczuk, Jenny; University of Oxford, National Perinatal Epidemiology Unit<br>Sullivan, Elizabeth; University of Technology Sydney Faculty of Health, Australian Centre for Public and Population Health Research<br>Knight, Marian; National Perinatal Epidemiology Unit
**Primary Subject Heading**:	Epidemiology
Secondary Subject Heading:	Obstetrics and gynaecology, Public health
Keywords:	Obesity, OBSTETRICS, obesity in pregnancy

SCHOLARONE™
Manuscripts

05/06/2018

A bi-national cohort study comparing the management and outcomes of pregnancy women with a BMI>50-59.9kg/m^2 and those with a BMI 60kg/m^2 or greater

Running title: The management, maternal and perinatal outcomes of women with a BMI 60kg/m^2 or greater

Stephen J. McCall[1]; Zhuoyang Li[2], Jennifer J. Kurinczuk[1], Elizabeth Sullivan[2]*, Marian Knight[1]*

[1] National Perinatal Epidemiology Unit (NPEU), Nuffield Department of Population Health, University of Oxford, Oxford, UK

[2]The Australian Centre for Public and Population Health Research, University of Technology Sydney, Sydney, Australia

*Joint senior authors

Address for correspondence: Stephen McCall, National Perinatal Epidemiology Unit, Nuffield Department of Population Health, University of Oxford, Richard Doll Building, Old Road Campus, Oxford, OX3 7LF, UK.

Email: Stephen.mccall@npeu.ox.ac.uk

Word count: 3223

For peer review only

05/06/2018

Abstract

Objectives: To compare the management, maternal and perinatal outcomes of women with a BMI 60kg/m^2 or greater with women with a BMI >50-59.9kg/m^2.

Design: International collaborative cohort study

Setting: Bi-national study in the UK and Australia

Participants: UK: all pregnant women and Australia: women gave birth (birthweight ≥400g or gestation ≥20 weeks)

Methods: Data from the Australasian Maternity Outcomes Surveillance System and UK Obstetric Surveillance System. Management, maternal and infant outcomes were compared between women with BMI 60kg/m^2 or greater and women with a BMI >50-59.9kg/m^2, using unconditional logistic regression.

Results: The sociodemographic characteristics and previous medical histories were similar between the 111 women with a BMI 60kg/m^2 or greater and the 821 women with a BMI >50-59.9kg/m^2. Women with a BMI 60kg/m^2 or greater had higher odds of thromboprophylaxis usage in both the antenatal (24% vs 12%; OR:2.25, 95%CI:1.39-3.64) and postpartum periods (78% vs 66%; OR:1.6, 95CI:1.04-2.70). Women with BMI 60kg/m^2 or greater had nearly double the odds of preeclampsia (adjusted OR:1.83 (95%CI:1.01-3.30)). No other maternal or perinatal outcomes were statistically significantly different. Severe adverse outcomes such as perinatal death were uncommon in both groups thus limiting the power of these comparisons. The rate of perinatal deaths was 18 per 1000 births for those with BMI 60kg/m^2 or greater ; 12.1 per 1000 births for those with BMI>50-59.9 kg/m^2; those with BMI 60kg/m^2 or greater had a non-significant increased odds of perinatal death (unadjusted OR:1.46, 95% CI:0.31-6.73).

For peer review only - http://bmjopen.bmj.com/site/about/guidelines.xhtml

05/06/2018

Conclusions: Women are managed differently on the basis of BMI even at this extreme as shown by thromboprophylaxis. The preeclampsia result suggests that future research should examine whether weight reduction of any amount prior to pregnancy could reduce poor outcomes even if women remain extremely obese.

Strengths

- Population based study examining extreme obesity using national data from the UK and Australia.
- International collaborative studies allow the examination of rare exposures.

Limitations

-This study lacked the power to examine many maternal and perinatal outcomes despite having data from two national studies

-Some outcomes were not comparable between Australia and the UK so could not be explored.

For peer review only

05/06/2018

Introduction

Obesity is a major risk factor for non-communicable disease and morbidity in later life. It has reached epidemic levels in many high-income settings across all age-ranges. Obesity is defined as a body mass index (BMI) of ≥30 kg/m^2. Increasing rates of obesity in the general population are associated with an increasing trend towards obesity in pregnancy [1] Within the general population, the largest increases in obesity have been in the highest BMI groups [2] and this is also true for extreme obesity in pregnancy [3].

Maternal obesity is a risk factor for a number of pregnancy related complications and its relationship with these complications are complex [4, 5]. These relationships can be partially explained through pre-existing comorbidities such as diabetes [6], hypertension [6, 7] and asthma [6]. Pre-existing comorbidities have been shown to increase the risk of preeclampsia [8, 9] and venous thromboembolic events [10]. However, there remain other mechanisms that explain the association between obesity and preeclampsia/ venous thromboembolism, to specify a few, these are inflammation [11], insulin resistance [12] and oxidative stress [13, 14].

There have been several studies investigating the prevalence, outcomes and managements of extreme obesity in pregnancy (BMI ≥50 kg/m^2) [15-17]. These have aimed to test whether there was a dose response relationship between increased BMI and complications of pregnancy. Within the extremely obese group (BMI ≥50 kg/m^2) women included have had a BMI ranging from ≥50 kg/m^2 to approximately 75 kg/m^2. Whilst it may be the case that the risks rise exponentially with BMI it is possible that above a certain BMI, the risks of maternal and perinatal complications as a result of obesity do not increase due to the competing risks of other comorbidities. This remains to be investigated, as current published data do not allow the division of women into the highest BMI groups.

For peer review only - http://bmjopen.bmj.com/site/about/guidelines.xhtml

05/06/2018

Previous research pooling together international data on rare exposures in pregnancy has been limited due to heterogeneity of definitions, methods and populations [18]. The obstetric surveillance systems in Australia and the UK were designed to be compatible with data collection using similar definitions with a view to pooling data. As a result, there are comparable data available to combine national studies, providing a large enough sample compare two groups of women within a cohort of extremely obese women. This study aimed to compare the characteristics, management including guideline adherence for prevention of venous thromboembolism, maternal and perinatal outcomes of women at the extremes of obesity.

For peer review only

05/06/2018

Methods

Study population and design

This study was an international population-based cohort study, using secondary analysis of two national cohort studies of extreme maternal obesity, which were undertaken in Australia and the United Kingdom [16, 19]. For the purposes of the analysis, the exposed cohort were those pregnant women who had a BMI 60kg/m^2 or greater and the unexposed comparison cohort were those with a BMI >50-59.9 kg/m^2. Woman were included in the study if they had a BMI >50 kg/m^2 at any point during pregnancy and were included as part of the respective national studies [16, 19].

Anonymous data were prospectively collected using each of the national obstetric surveillance system the United Kingdom Obstetric Surveillance System (UKOSS) or Australasian Maternity Outcomes Surveillance System (AMOSS). The methods of each system have been described elsewhere in detail [19-21]. Briefly, in the UK, nominated reporters within each consultant-led obstetric unit received a monthly mailing card; the card had a tick box to indicate whether there had been a case of extreme obesity that month. There was also a box to indicate that there were no cases. Reporters returned cards regardless of whether there had been a case of extreme obesity. When a case was notified the reported received a data collection form. Using the medical records of the patient, information on demographic characteristics, obstetric history, medical history (including height and weight), management and outcomes were collected.

A similar method was used to identify women with extreme obesity in Australia. Designated reporters within each participating maternity unit within Australia were sent a monthly email. The reporter either responded with a “case” or a “nil case” to indicate whether there had truly been no cases. Once a case was reported, the reporter entered data on an online data

For peer review only - http://bmjopen.bmj.com/site/about/guidelines.xhtml

collection form using the case notes of the woman. The AMOSS system had 66% coverage of all women giving birth in Australia during the study [19].

Outcomes, management and potential covariates relevant to the research question were identified from the literature. On the basis of this, possible covariates and outcomes were identified in the respective UKOSS and AMOSS datasets. Each variable was mapped between the AMOSS and UKOSS datasets and an assessment of the comparability was made. On occasions, where the coding differed, harmonisation of the coding was devised and applied. This resulted in uniform values and labels of variables across both datasets. An assessment was made to determine whether the variables were measuring the same clinical phenotype in similar ways.

The covariates explored in the analysis were age, smoking status during pregnancy, previous pregnancy problems, pre-existing medical problems, pre-existing hypertension, parity and multiple pregnancies.

The missing data in similar datasets has been shown not to be missing at random; as a result multiple imputation was not considered appropriate [22]. A missing category was this created for each variable to account for the missing data. Primarily, complete case analysis was used in the multivariable analysis and a sensitivity analysis including the missing categories was used to assess the impact of missing data on the point estimates.

The sample size was predetermined by the size of the existing studies; therefore the sample was fixed at 111 women who had a BMI 60kg/m^2 or greater and 821 women who had a BMI >50-59.9kg/m^2. For the lowest frequency outcome (perinatal death), which had an incidence of 1.2% in the unexposed group, given the sample size the minimum odds ratio detectable as

For peer review only - http://bmjopen.bmj.com/site/about/guidelines.xhtml

statistically significant with 80% power at the 5% significance level was 5.63. For the highest frequency outcome, which had an incidence of 66.4% (thromboprophylaxis postnatally) in the unexposed group, the minimum odds ratio detectable as statistically significant with 80% power at the 5% significance level was 1.99.

Statistical analysis

Descriptive analyses were undertaken using the Chi Square test or Wilcoxon rank sum test as appropriate. These analyses assessed whether there was a statistical difference in characteristics between those women who had a BMI 60kg/m^2 or greater and those with a BMI >50-59 kg/m^2.

Each outcome was individually modelled in a univariable analysis using unconditional logistic regression, with results presented as unadjusted odds ratios (uOR) with 95% confidence intervals (95% CI). The exposure variable in each model was extreme obesity BMI 60kg/m^2 or greater. To account for clustering of infants within mothers' (multiple births) robust estimates of variance were calculated. Collinearity was assessed between all plausible linear associations prior to multivariable analysis, using Pearson's correlation coefficient.

Only outcomes that were statistically significant at the univariable level were included in the multivariable analysis. In the multivariable analysis, potential explanatory variables were sequentially added to the univariable model in a forward stepwise method with an examination of the results as each variable was added. A plausible explanatory variable was included in the final model if it was associated with the exposure and outcome (P-value for Wald test<0.05) or significantly improved the model fit assessed by likelihood ratio tests at the 5% significance level. Statistical analysis was completed using STATA V.13 (STATA CORP, Texas, USA).

For peer review only - http://bmjopen.bmj.com/site/about/guidelines.xhtml

A post hoc analysis was completed to assess the risk factors for venous thromboembolism possessed by those who did not receive postnatal thromboprophylaxis. This was a country specific analysis using risk factors of venous thromboembolism which were identified from the RCOG and South Australian Maternal & Neonatal Clinical Network guidelines [23, 24].

Patient and Public Involvement statement

There is PPI involvement in the UKOSS steering committee through lay members. The UKOSS steering committee assisted in the study design and management of the study. The AMOSS advisory group has PPI involvement through consumer, Maori and Pacific and Aboriginal and Torres Strait Islander members. The AMOSS advisory group provides advice on the implementation, delivery and development of the AMOSS system. The group also assists with the translation of findings into practice.

Ethics committee approval

The Australian collaborators obtained approval for the study from the NSW Population and Health Services Research Ethics Committee and multiple Human Research Ethics Committees across Australia [25]. Ethics committee approval for secondary analysis of anonymous UK data was not required.

Results

During the period September 2007-August 2008, 617 women with a BMI>50 kg/m^2 were identified through the UK Obstetric Surveillance System. Between January - October 2010, 315 women with a BMI>50 kg/m^2 were identified using the Australasian Maternity Outcomes Surveillance System. Overall there were 111 women with a BMI 60kg/m^2 or greater and 821 women with a BMI >50-59.9kg/m^2.

For peer review only - http://bmjopen.bmj.com/site/about/guidelines.xhtml

05/06/2018

Women with a BMI 60kg/m^2 or greater were slightly older, the sociodemographic characteristics and previous medical histories were otherwise similar the two groups of women (Table 1).

A high proportion in both groups experienced difficulties in visualisation of ultrasound (70.3% vs. 65.7%) although this was not statistically significant between the groups. Fewer women in both groups received antenatal thromboprophylaxis (24.3% BMI 60kg/m^2 or greater and 12.3% >50-59.9kg/m^2) compared to postnatal thromboprophylaxis (77.5% and 66.4%) (Table 2). Women with a BMI 60kg/m^2 or greater had a significantly higher odds of preeclampsia (uOR: 1.91 (95%CI: 1.08-3.39)), and of receiving either thromboprophylaxis antenatally (uOR:2.25 (95%CI:1.39-3.64)) or postnatally (uOR: 1.68 (95%CI: 1.04-2.70)) compared to those with a BMI >50-59.9kg/m^2 (Table 2). Supplementary tables 1 and 2 show that 27% and 32% of women should have received thromboprophylaxis postnatally in the UK and Australia as they had the relevant risk factors for it to be indicated , respectively.

Although not statistically significant, a higher proportion of women with a BMI 60kg/m^2 or greater experienced other adverse outcomes other than preeclampsia/eclampsia. Preeclampsia/eclampsia was examined in a multivariable model. The presence of a BMI 60kg/m^2 or greater was associated with a two-fold increase in the odds of having preeclampsia/eclampsia (aOR:1.83 (95%CI: 1.01-3.30)) compared to those with a BMI >50-59kg/m^2, after adjusting for smoking status, pre-existing diabetes and parity. The results of the proxy variable model did not materially differ to those of the complete case analysis.

Severe adverse outcomes such as perinatal death were uncommon in both groups (n=2 (18 per 1000 births), BMI 60kg/m^2 or greater vs. n=10 (12 per 1000 births), BMI ≥ 50-59kg/m^2). There were no statistically significant differences in perinatal outcomes between both obesity groups (see table 3).

For peer review only - http://bmjopen.bmj.com/site/about/guidelines.xhtml

05/06/2018

Discussion

Main Findings

Compared with women with a BMI >50-59 women with a BMI 60kg/m^2 or greater had an increased risk of preeclampsia/eclampsia, There were very few statistically significant differences in outcomes between the two very high BMI groups. Nevertheless, the direction of effects favours the lower BMI group for most outcomes. Further research should test whether any weight reduction could reduce poor outcomes even if women remain extremely obese. Importantly, the perinatal mortality rate was higher in both groups compared with both the UK/Australian rate. Women are being managed differently on the basis of BMI even at this extreme as use of thromboprophylactic drugs varied between the two high BMI groups.

Strengths and limitations

Both prospective population based surveillance systems use a robust methodology, which reduces the risk of selection bias. Two national studies allowed the examination of women with a BMI 60kg/m^2 or greater in a high resource setting and thus overcomes some of the limitations of previous research which was limited by the number of women in the extreme ends of the BMI distribution. Nevertheless, despite pooling of national data the number of women in each group was still relatively small, which limited the study power, particularly when investigating rare outcomes.

This study did not have access to ethnicity from Australia and socioeconomic measures were not comparable between the countries. Thus the adjusted odd ratio presented may be vulnerable to residual confounding if ethnicity and socioeconomic status were associated with both the outcome and exposure.

For peer review only

05/06/2018

This analysis aimed only to compare the pregnancy outcomes of two groups of extremely obese women, and does not therefore provide any information on the outcomes of these extremely obese pregnant in comparison to pregnant women with BMIs within the normal range. Comparisons with pregnant women who have a lower BMI have been previously published separately [16,19].

Interpretation

One of the novel benefits of this multi-national study was the ability to examine a subset of the more extreme end of the spectrum of obesity. This demonstrated that women with a BMI 60kg/m^2 or greater had very similar characteristics and experienced similar management compared to women with a BMI>50 -<60 kg/m^2. Interestingly, the BMI 60kg/m^2 or greater group had an increased risk of preeclampsia/eclampsia; which supports the hypothesis of a 'dose response' relationship between obesity and preeclampsia/eclampsia seen at lower BMIs [26] and super obesity [17, 27].

The comparison of extreme maternal obesity and a representative BMI group has been previously studied [16, 19]. The risk of preeclampsia, venous thromboembolism, preterm delivery, shoulder dystocia, caesarean delivery was elevated in women with extreme maternal obesity compared to non-extremely obese women [16, 19]. Despite few statistically significant differences in outcomes between the two groups, the literature highlights that the risk is substantially higher for extremely obese women compared to women in a normal BMI group.

The BMI 60kg/m^2 or greater cohort had a higher proportion of perinatal deaths and stillbirths than the BMI >50-59.9 kg/m^2 cohort, although these were not statistically significantly different possibly because of the small numbers involved. The absolute rate of perinatal death for the 60kg/m^2 or greater cohort was three times higher than the UK rate (5.6 per 1000

For peer review only - http://bmjopen.bmj.com/site/about/guidelines.xhtml

births) and 2.5 times higher than the Australian rate (7.3 per 1000); while the rate of perinatal mortality in the >50-59.9 kg/m^2 cohort was just over twice that of the UK rate and was 1.5 times higher than the Australian perinatal mortality rate [28, 29].

A previous study of extreme obesity that examine perinatal outcomes has suggested that there is a dose response relationship between BMI and perinatal outcomes [17]. The small sample size and relative rarity of adverse perinatal outcomes in this analysis did not allow the role of chance to be excluded for most outcomes even with pooling two national studies.

The results of this study show that the degree of relative obesity impacted on thromboprophylaxis practice. The Royal College of Obstetricians and Gynaecologists' guideline states that any women with a BMI >40kg/m^2 should be considered at intermediate risk of venous thromboembolism and should be given at least 10 days of thromboprophylaxis postnatally [23]. Within Australia there is regional variation in the guidelines concerning BMI and postpartum thromboprophylaxis. The Queensland state guideline suggests that a woman must possess three or more risk factors (BMI >30 kg/m^2 being one of these risk factors) to be given low molecular weight heparin for 6 days postnatally, while the South Australian government and current expert opinion recommends that BMI ≥30 kg/m^2 plus one major risk factor for thromboembolism requires prophylactic anticoagulation for 5 days postpartum [24, 30]. Data from this study suggests guidelines appear to be followed variably due to the large variation in practice between the two cohorts. This suggests more implementation work within clinical settings is needed to help these guidelines be followed. Nevertheless, the results show that BMI has an important impact on clinical decisions concerning the administration of thromboprophylaxis postnatally.

Importantly, approximately 75% had postnatal thromboprophylaxis which is smaller than expected considering this was an extremely obese population. Nearly a third of women in

For peer review only - http://bmjopen.bmj.com/site/about/guidelines.xhtml

both countries had the appropriate risk factors to indicate the use of thromboprophylaxis postnatally. This highlights an important area for improvement of clinical practice to prevent a potentially fatal venous thromboembolism.

Interestingly, there were no venous thromboembolic events in the BMI 60kg/m^2 or greater group, which was the group in which the larger proportion of women received thromboprophylaxis although again these are very rare events. Previous studies have shown that BMI is a strong risk factor of venous thromboembolism [31, 32] and the risk is amplified in those who have a high BMI and were immobilized [33].

This study was a secondary data analysis of women identified during 2008 in the UK and 2010 in the Australia. As a result, it is likely that the proportion of women who have a BMI >50 since the original studies is likely to be much larger which makes the findings of this study even more pertinent.

Conclusions

There were very few statistically significant differences in outcomes between these two high BMI groups. However, the direction of effect favours the lower BMI group for most outcomes and a type II error cannot be excluded given the small number of outcomes. Preeclampsia risk is increased with increasing BMI in the morbidly obese women. Further research should test whether any weight reduction could reduce poor outcomes even if women remain extremely obese. Women are clearly being managed differently on the basis of BMI even at this extreme as shown by the thromboprophylaxis data. Furthermore, there was a failure to full apply thromboprophylaxis guidelines fully in 2007-2008 which emphasises a need to ensure women at risk of venous thromboembolism receive appropriate prevention care.

For peer review only - http://bmjopen.bmj.com/site/about/guidelines.xhtml

Acknowledgements: We thank the AMOSS advisory group and UKOSS steering committee for their input into the respective obesity studies. Dr Manisha Nair and Dr Bryn Kemp for their advice on the project.

Data sharing statement: The NPEU Data sharing agreement can be found here: https://www.npeu.ox.ac.uk/downloads/files/npeu/policies/Data%20Sharing%20Policy.pdf Access to the Australian data must be made to the AMOSS steering committee.

Disclosures of interest: None

Funding: SM is funded by the Nuffield Department of Population Health and Medical Research Council (MRC) training grant MR/K501256/1. Marian Knight is funded by an NIHR Research Professorship. The views expressed in this publication are those of the author(s), and not necessarily those of the MRC, the NHIR or the Department of Health. The funder had no role in the study design, data collection and analysis, decision to publish, or preparation of the article. The National Health and Medical Research Council Project Grant (Application 510298) for funding The Australian Maternity Outcomes Surveillance System: Improving safety and quality of maternity care in Australia (AMOSS) from 2008–2012.

Author contribution: MK, JK, ES conceived the study. MK, JK, ES and SM designed the study. ZL extracted the Australian data. SM analysed the data and wrote the first draft. All authors interpreted the data and edited the manuscript.

Tables list

Table 1. Sociodemographic characteristics and previous medical problems in women with BMI 60kg/m^2 or greater and comparison women (BMI >50-59.9kg/m^2).

Table 2. Maternal outcomes and management in women with BMI 60kg/m^2 or greater and comparison women (BMI >50-59.9kg/m^2).

Table 3. Perinatal outcomes in women with BMI 60kg/m^2 or greater and comparison women (BMI >50-59.9kg/m^2).

For peer review only - http://bmjopen.bmj.com/site/about/guidelines.xhtml

05/06/2018

References

1. HESLEHURST, N., RANKIN, J., WILKINSON, J. R. & SUMMERBELL, C. D. 2010. A nationally representative study of maternal obesity in England, UK: Trends in incidence and demographic inequalities in 619 323 births, 1989-2007. *International Journal of Obesity,* 34**,** 420-428.
2. STURM, R. 2007. Increases in morbid obesity in the USA: 2000–2005. *Public Health,* 121**,** 492-496.
3. KIM, S. Y., DIETZ, P. M., ENGLAND, L., MORROW, B. & CALLAGHAN, W. M. 2007. Trends in Pre□pregnancy Obesity in Nine States, 1993–2003. *Obesity,* 15**,** 986-993.
4. BHATTACHARYA, S., CAMPBELL, D. M., LISTON, W. A. & BHATTACHARYA, S. 2007. Effect of body mass index on pregnancy outcomes in nulliparous women delivering singleton babies. *BMC public Health,* 7**,** 168.
5. CATALANO, P. M. & EHRENBERG, H. M. 2006. Review article: The short- and long-term implications of maternal obesity on the mother and her offspring. *BJOG: An International Journal of Obstetrics & Gynaecology,* 113**,** 1126-1133.
6. MOKDAD, A.H., FORD, E.S., BOWMAN, B.A., DIETZ, W.H., VINICOR, F., BALES, V.S. and MARKS, J.S., 2003. Prevalence of obesity, diabetes, and obesity-related health risk factors, 2001. *JAMA,* 289(1), pp.76-79.
7. RE, R.N., 2009. Obesity-related hypertension. *The Ochsner Journal,* 9(3), pp.133-136.
8. DUCKITT, K. and HARRINGTON, D., 2005. Risk factors for pre-eclampsia at antenatal booking: systematic review of controlled studies. *BMJ,* 330(7491), p.565.
9. SIBIA, B.M., Gordon, T., Thom, E., Caritis, S.N., Klebanoff, M., McNellis, D. and Paul, R.H., 1995. Risk factors for preeclampsia in healthy nulliparous women: a prospective multicenter study. *American Journal of Obstetrics & Gynecology,* 172(2), pp.642-648.
10. JAMES, A.H., JAMISON, M.G., BRANCAZIO, L.R. and MYERS, E.R., 2006. Venous thromboembolism during pregnancy and the postpartum period: incidence, risk factors, and mortality. *American Journal of Obstetrics & Gynecology,* 194(5), pp.1311-13.
11. WOLF, M., KETTYLE, E., SANDLER, L., ECKER, J. L., ROBERTS, J. & THADHANI, R. 2001. Obesity and preeclampsia: the potential role of inflammation. *Obstetrics & Gynecology,* 98**,** 757-762.
12. VAN GAAL, L. F., MERTENS, I. L. & CHRISTOPHE, E. 2006. Mechanisms linking obesity with cardiovascular disease. *Nature,* 444**,** 875.
13. ROBERTS, J. M., BODNAR, L. M., PATRICK, T. E. & POWERS, R. W. 2011. The role of obesity in preeclampsia. *Pregnancy Hypertension: An International Journal of Women's Cardiovascular Health,* 1**,** 6-16.
14. FREEDMAN, J. E. 2008. Oxidative stress and platelets. *Arteriosclerosis, thrombosis, and vascular biology,* 28**,** s11-s16.

For peer review only - http://bmjopen.bmj.com/site/about/guidelines.xhtml

05/06/2018

15. MARTIN, A., KRISHNA, I., ELLIS, J., PACCIONE, R. & BADELL, M. 2014. Super obesity in pregnancy: difficulties in clinical management. *Journal of Perinatology,* 34**,** 495-502.
16. KNIGHT, M., KURINCZUK, J. J., SPARK, P., BROCKLEHURST, P. & SYSTEM, U. K. O. S. 2010. Extreme obesity in pregnancy in the United Kingdom. *Obstetrics & Gynecology,* 115**,** 989-97.
17. MARSHALL, N. E., GUILD, C., CHENG, Y. W., CAUGHEY, A. B. & HALLORAN, D. R. 2012. Maternal superobesity and perinatal outcomes. *American Journal of Obstetrics & Gynecology,* 206**,** 417.e1-6.
18. KNIGHT, M., CALLAGHAN, W. M., BERG, C., ALEXANDER, S., BOUVIER-COLLE, M. H., FORD, J. B., JOSEPH, K. S., LEWIS, G., LISTON, R. M., ROBERTS, C. L., OATS, J. & WALKER, J. 2009. Trends in postpartum hemorrhage in high resource countries: a review and recommendations from the International Postpartum Hemorrhage Collaborative Group. *BMC Pregnancy & Childbirth,* 9**,** 55.
19. SULLIVAN, E. A., DICKINSON, J. E., VAUGHAN, G. A., PEEK, M. J., ELLWOOD, D., HOMER, C. S. E., KNIGHT, M., MCLINTOCK, C., WANG, A., POLLOCK, W., JACKSON PULVER, L., LI, Z., JAVID, N., DENNEY-WILSON, E. & CALLAWAY, L. 2015. Maternal super-obesity and perinatal outcomes in Australia: A national population-based cohort study. *BMC Pregnancy and Childbirth,* 15:322
20. KNIGHT, M., KURINCZUK, J. J., TUFFNELL, D. & BROCKLEHURST, P. 2005. The UK Obstetric Surveillance System for rare disorders of pregnancy. *BJOG: An International Journal of Obstetrics and Gynaecology,* 112**,** 263-265.
21. MCDONNELL, N., KNIGHT, M., PEEK, M. J., ELLWOOD, D., HOMER, C. S. E., MCLINTOCK, C., VAUGHAN, G., POLLOCK, W., LI, Z., JAVID, N. & SULLIVAN, E. 2015. Amniotic fluid embolism: an Australian-New Zealand population-based study. *BMC Pregnancy and Childbirth,* 15**,** 352.
22. LINDQUIST, A., KNIGHT, M. & KURINCZUK, J. J. 2013. Variation in severe maternal morbidity according to socioeconomic position: a UK national case–control study. *BMJ open,* 3**,** e002742.
23. ROYAL COLLEGE OF OBSTETRICS AND GYNAECOLOGY (RCOG) 2015. Reducing the Risk of Venous Thromboembolism during Pregnancy and the Puerperium. Green-top Guideline No. 37a. London: RCOG.
24. SOUTH AUSTRALIAN MATERNAL & NEONATAL CLINICAL NETWORK. 2013. South Australian Perinatal Practice Guidelines: thromboprophylaxis and thromboembolic disease in pregnancy. Department of Health, Government of South Australia.
25. VAUGHAN, G., POLLOCK, W., PEEK, M. J., KNIGHT, M., ELLWOOD, D., HOMER, C. S., PULVER, L. J., MCLINTOCK, C., HO, M. T. & SULLIVAN, E. A. 2012. Ethical issues: The multi-centre low-risk ethics/governance review process and AMOSS. *Australian and New Zealand Journal of Obstetrics and Gynaecology,* 52**,** 195-203.
26. BODNAR, L. M., CATOV, J. M., KLEBANOFF, M. A., NESS, R. B. & ROBERTS, J. M. 2007. Prepregnancy body mass index and the occurrence of severe hypertensive disorders of pregnancy. *Epidemiology,* 18**,** 234-239.
27. MBAH, A. K., KORNOSKY, J. L., KRISTENSEN, S., AUGUST, E. M., ALIO, A. P., MARTY, P. J., BELOGOLOVKIN, V., BRUDER, K. & SALIHU, H. M. 2010. Super-obesity and risk for early and late pre-eclampsia. *BJOG: An International Journal of Obstetrics and Gynaecology,* 117**,** 997-1003.

For peer review only

For peer review only - http://bmjopen.bmj.com/site/about/guidelines.xhtml

05/06/2018

28. MANKTELOW, B., SMITH, L., PRUNET, C., SMITH, P., BOBY, T., HYMAN-TAYLOR, P., KURINCZUK, J., FIELD, D., E., D. & ON BEHALF OF THE MBRRACE-UK COLLABORATION 2017. *MBRRACE-UK Perinatal Mortality Surveillance Report: UK Perinatal Deaths for Births from January to December 2015*, Department of Health Sciences, University of Leicester.

29. MONK, A., HARRIS, K., DONNOLLEY, N., HILDER, L., HUMPHREY, M., GORDON, A. & CHAMBERS, G on behalf of Austrailian Institute of Health and Welfare.. 2016. Perinatal deaths in Australia, 1993–2012. Perinatal deaths. Canberra: Austrailian Institute of Health and Welfare.

30. MCLINTOCK, C., BRIGHTON, T., CHUNILAL, S., DEKKER, G., MCDONNELL, N., MCRAE, S., MULLER, P., TRAN, H., WALTERS, B. N. & YOUNG, L. 2012. Recommendations for the prevention of pregnancy□associated venous thromboembolism. *Australian and New Zealand Journal of Obstetrics and Gynaecology,* 52**,** 3-13.

31. LARSEN, T. B., SØRENSEN, H. T., GISLUM, M. & JOHNSEN, S. P. 2007. Maternal smoking, obesity, and risk of venous thromboembolism during pregnancy and the puerperium: A population-based nested case-control study. *Thrombosis Research,* 120**,** 505-509.

32. KNIGHT, M. 2008. Antenatal pulmonary embolism: risk factors, management and outcomes. *BJOG: An International Journal of Obstetrics & Gynaecology,* 115**,** 453-461.

33. JACOBSEN, A. F., SKJELDESTAD, F. E. & SANDSET, P. M. 2008. Ante- and postnatal risk factors of venous thrombosis: a hospital-based case–control study. *Journal of Thrombosis and Haemostasis,* 6**,** 905-912.

For peer review only

05/06/2018

Table 1. Sociodemographic characteristics and previous medical problems in women with BMI 60kg/m^2 or greater and comparison women (BMI >50-59.9kg/m^2).

Characteristic		Number (%) of obese women BMI 60 or greater (n=111)		Number (%) of women BMI 50 - 59.9 (n=821)		p-value
Sociodemographic characteristics						
Age	*Mean (Std)*	31.7	(5.51)	30.3	(5.67)	0.017
BMI at booking	*Median (IQR)*	61.7	(60-64.9)	52.3	(50.8-54.9)	<0.001
Max recorded BMI	*Median (IQR)*	62.9	(61-66.8)	52.7	(50.9-55.0)	<0.001
Smoking status	*Never/ex-smoker*	85	(76.6)	599	(73)	0.42
	Smoked during pregnancy	24	(21.6)	206	(25.1)	
	Missing	2	(1.8)	16	(1.9)	
Known previous medical history						
Previous pregnancy problems	*None*	41	(36.9)	273	(33.3)	0.713
	Yes	33	(29.7)	266	(32.4)	
	Not applicable	35	(31.5)	271	(33.0)	
	Missing	2	(1.8)	11	(1.3)	
Known cardiac disease	*None*	109	(98.2)	812	(98.9)	0.174
	Yes	2	(1.8)	5	(0.6)	
	Missing	0	(0)	4	(0.5)	
Known renal disease	*None*	110	(99.1)	809	(98.5)	0.937
	Yes	1	(0.9)	8	(1.0)	
	Missing	0	(0)	4	(0.5)	
Known mental health issues	*None*	99	(89.2)	756	(92.1)	0.219
	Yes	12	(10.8)	61	(7.4)	
	Missing	0	(0)	4	(0.5)	
Known asthma	*None*	98	(88.3)	720	(87.7)	0.961
	Yes	13	(11.7)	97	(11.8)	
	Missing	0	(0)	4	(0.5)	
Previous caesarean delivery	*None*	53	(47.7)	366	(44.6)	0.297
	Yes	22	(19.8)	181	(22.0)	
	Not applicable	35	(31.5)	271	(33.0)	
	Missing	1	(0.9)	3	(0.4)	
Parity	*Nulliparous*	35	(31.5)	271	(33)	0.803
	Multiparous	75	(67.6)	550	(67)	
	Missing	1	(0.9)	0	(0)	
Current pregnancy						

For peer review only

05/06/2018

Multiple pregnancy	*Singleton*	108	(97.3)	800	(97.4)	0.928
	Twin pregnancy	3	(2.7)	21	(2.6)	
Known hypertension prior to pregnancy requiring treatment	*None*	103	(92.8)	767	(93.4)	0.693
	Yes	8	(7.2)	51	(6.2)	
	Missing	0	(0)	3	(0.4)	
Known pre-existing diabetes prior to pregnancy	*None*	101	(91.0)	757	(92.2)	0.657
	Yes	10	(9.0)	64	(7.8)	
	Missing	-	-	-	-	
Insulin dependent diabetes	*Yes*	4	(3.6)	17	(2.1)	0.663

For peer review only

05/06/2018

Table 2. Maternal outcomes and management in women with BMI 60kg/m^2 or greater and comparison women (BMI >50-59.9kg/m^2).

		Number (%) of women BMI 60 or greater (n=111)		Number (%) of women BMI >50-59.9(n=821)		Unadjusted odds ratio	95% Confidence interval	P-value
Management								
Difficulties undertaking ultrasounds	*No*	30	(27)	228	(27.8)	1		
	Yes	78	(70.3)	539	(65.7)	1.10	(0.70-1.72)	0.678
	Missing	3	(2.7)	54	(6.6)			
Induced	*No*	59	(53.2)	405	(49.3)	1		
	Yes	40	(36)	303	(36.9)	0.91	(0.59-1.39)	0.652
	Missing	12	(10.8)	113	(13.8)			
Syntocinon	*No*	25	(22.5)	243	(29.6)	1		
	Yes	41	(36.9)	289	(35.2)	1.38	(0.82-2.33)	0.231
	Missing	45	(40.5)	289	(35.2)			
Caesarean delivery	*No*	48	(43.2)	398	(48.5)	1		.
	Yes	62	(55.9)	411	(50.1)	1.25	(0.84,1.87)	0.274
	Missing	1	(0.9)	12	(1.5)			
Thromboprophylaxis usage antenatal	*No*	84	(75.7)	706	(86)	1		
	Yes	27	(24.3)	101	(12.3)	2.25	(1.39-3.64)	0.001
	Missing	0	(0)	14	(1.7)			
Thromboprophylaxis post	*No*	24	(21.6)	255	(31.1)	1		
	Yes	86	(77.5)	545	(66.4)	1.68	(1.04-2.70)	0.033
	Missing	1	(0.9)	21	(2.6)			
Maternal Outcome								
Wound infection in those with caesarean	*No*	47	(42.3)	344	(41.9)	1		
	Yes	14	(12.6)	57	(6.9)	1.80	(0.93-3.48)	0.081
	N/A	49	(44.1)	410	(49.9)			
	Missing	1	(0.9)	10	(1.2)			
Venous thromboembolism *	*No*	111	(100)	807	(98.3)			
	Yes	0	(0)	7	(0.9)	0	(0.0-4.00)	0.325
	Missing	0	(0)	7	(0.9)			
Hypertensive disorder during pregnancy	*No*	77	(69.4)	631	(76.9)	1		
	Yes	33	(29.7)	183	(22.3)	1.48	(0.95-2.29)	0.082
	Missing	1	(0.9)	7	(0.9)			
Pregnancy induced hypertension	*No*	94	(84.7)	702	(85.5)	1		
	Yes	16	(14.4)	112	(13.6)	1.07	(0.61-1.88)	0.823

For peer review only

05/06/2018

	Missing	1	(0.9)	7	(0.9)			
Pre-eclampsia/eclampsia	*No*	93	(83.8)	743	(90.5)	1		
	Yes	17	(15.3)	71	(8.6)	1.91	(1.08-3.39)	0.026
	Missing	1	(0.9)	7	(0.9)			

For peer review only

5 June 18

Table 3. Perinatal outcomes in women with BMI 60kg/m^2 or greater and comparison women (BMI >50-59.9kg/m^2).

		Number (%) of women BMI 60 or greater		Number (%) of women BMI >50-59.9		Unadjusted odds ratio	95% CI	P-Value
Perinatal death*	*No*	112	(98.2)	815	(98.5)	1		
	Yes	2	(1.8)	10	(1.2)	1.46	(0.31-6.74)	0.631
	Missing	0	(0)	2	(0.2)			
Still birth >24weeks gestation*	*No*	112	(98.2)	818	(98.9)	1		
	Yes	2	(1.8)	7	(0.8)	2.09	(0.43-10.19)	0.363
	Missing	0	(0)	2	(0.2)			
Preterm birth	*No*	101	(90.2)	730	(89)	1		
	Yes	10	(8.9)	87	(10.6)	0.83	(0.36-1.94)	0.668
	Missing	1	(0.9)	3	(0.4)			
Very preterm birth	*No*	111	(99.1)	804	(98)		Omitted	
	Yes	0	(0)	17	(1.6)			
	Missing	1	(0.9)	3	(0.4)			
Birthweight	*Mean (Std)*	3683.0	(752.1)	3603.7	(715.0)		Omitted	
Macrosomia (>4500grams)	*No*	98	(87.5)	746	(91.0)	1		
	Yes	14	(12.5)	72	(8.8)	1.48	(0.80-2.74)	0.211
	Missing	0	(0)	2	(0.2)			
Shoulder dystocia	*No*	44	(39.3)	373	(45.5)	1		
	Yes	1	(0.9)	19	(2.3)	0.45	(0.06-3.42)	0.438
	Missing	67	(59.8)	428	(52.5)			
Congenital abnormality	*No*	107	(95.5)	797	(97.2)	1		
	Yes	3	(2.7)	13	(1.6)	1.72	(0.48-6.14)	0.404
	Missing	2	(1.8)	10	(1.2)			
Infant respiratory problem	*No*	109	(97.3)	797	(97.2)	1		
	Yes	3	(2.7)	18	(2.2)	1.22	(0.35-4.21)	0.755
	Missing	0	(0)	5	(0.6)			
Apgar score <7 @ 5min	*No*	105	(93.8)	778	(94.9)	1		
	Yes	2	(1.8)	25	(3.0)	0.59	(0.14-2.54)	0.482
	Missing	5	(4.5)	17	(2.1)			

Odds ratios estimated using robust standard errors. *Denominator is birth (including multiple births) and stillbirths n=941. Denominator in the remainder of the table is live births (including multiple births).

For peer review only

For peer review only

For peer review only - http://bmjopen.bmj.com/site/about/guidelines.xhtml

8 December 17

Supplementary table 1. Risk factors and administration of postnatal thromboprophylaxis in UK population: Green Top guideline no. 37a. Royal College of Obstetrics and Gynaecology

	Did not receive thromboprophylaxis		Received postnatal thromboprophylaxis	
Only risk factor BMI ≥40	7	(4.2)	3	(0.7)
Two or more risk factors	160	(95.8)	432	(99.3)
Should have received thromboprophylaxis**	**167**	**(27.7)**	435	(72.2)

*These included: caesarean section, age ≥35, infection, parity ≥3, smoker, preeclampsia, caesarean section, multiple births and stillbirth (Other risk factors were in the RCOG guideline that were not available in this dataset).

**Row percentage

Supplementary table 2. Administration for postnatal thromboprophylaxis and criteria for guideline in Australia

	Did not receive thromboprophylaxis		Received thromboprophylaxis	
Meet the guideline criteria	109	97.3	192	97.7
Did not meet the guideline criteria	3	2.7	4	2
Should have received thromboprophylaxis***	**109**	**(36.2)**	192	63.8

*Major risk factors included: caesarean section, preeclampsia, infection and BMI ≥35kg/m^2

**Minor risk factors included: Age > 35 years, smoker, post-partum haemorrhage and parity ≥3 (Other risk factors were in the guideline that were not available in this dataset).

***Row percentage

Summary for indication for post-partum prophylactic anticoagulation for South Australian Perinatal Practice Guidelines thromboprophylaxis and thromboembolic disease in pregnancy by South Australian Maternal & Neonatal Clinical Network

-Emergency caesarean section OR 2 or more major risk factors

-At least one major and 2 or more minor risk factors

For peer review only

STROBE 2007 (v4) checklist of items to be included in reports of observational studies in epidemiology*
Checklist for cohort, case-control, and cross-sectional studies (combined)

Section/Topic	Item #	Recommendation	Reported on page #
**Title and abstract**	1	(*a*) Indicate the study's design with a commonly used term in the title or the abstract	1
		(*b*) Provide in the abstract an informative and balanced summary of what was done and what was found	2
**Introduction**			
Background/rationale	2	Explain the scientific background and rationale for the investigation being reported	4
Objectives	3	State specific objectives, including any pre-specified hypotheses	5
**Methods**			
Study design	4	Present key elements of study design early in the paper	6
Setting	5	Describe the setting, locations, and relevant dates, including periods of recruitment, exposure, follow-up, and data collection	6
Participants	6	(*a*) *Cohort study*—Give the eligibility criteria, and the sources and methods of selection of participants. Describe methods of follow-up<br>*Case-control study*—Give the eligibility criteria, and the sources and methods of case ascertainment and control selection. Give the rationale for the choice of cases and controls<br>*Cross-sectional study*—Give the eligibility criteria, and the sources and methods of selection of participants	6
		(*b*) *Cohort study*—For matched studies, give matching criteria and number of exposed and unexposed<br>*Case-control study*—For matched studies, give matching criteria and the number of controls per case	
Variables	7	Clearly define all outcomes, exposures, predictors, potential confounders, and effect modifiers. Give diagnostic criteria, if applicable	6
Data sources/ measurement	8*	For each variable of interest, give sources of data and details of methods of assessment (measurement). Describe comparability of assessment methods if there is more than one group	*6-7 not reported in detail*
Bias	9	Describe any efforts to address potential sources of bias	6 not reported in detail
Study size	10	Explain how the study size was arrived at	7
Quantitative variables	11	Explain how quantitative variables were handled in the analyses. If applicable, describe which groupings were chosen and why	7-8
Statistical methods	12	(*a*) Describe all statistical methods, including those used to control for confounding	8
		(*b*) Describe any methods used to examine subgroups and interactions	8
		(*c*) Explain how missing data were addressed	7

For peer review only

		(*d*) *Cohort study*—If applicable, explain how loss to follow-up was addressed<br>*Case-control study*—If applicable, explain how matching of cases and controls was addressed<br>*Cross-sectional study*—If applicable, describe analytical methods taking account of sampling strategy	
		(*e*) Describe any sensitivity analyses	
**Results**			
Participants	13*	(a) Report numbers of individuals at each stage of study—eg numbers potentially eligible, examined for eligibility, confirmed eligible, included in the study, completing follow-up, and analysed	10
		(b) Give reasons for non-participation at each stage	N/A
		(c) Consider use of a flow diagram	N/A
Descriptive data	14*	(a) Give characteristics of study participants (eg demographic, clinical, social) and information on exposures and potential confounders	10
		(b) Indicate number of participants with missing data for each variable of interest	10-11, Tables
		(c) *Cohort study*—Summarise follow-up time (eg, average and total amount)	
Outcome data	15*	*Cohort study*—Report numbers of outcome events or summary measures over time	10-11, Tables
		Case-control study—Report numbers in each exposure category, or summary measures of exposure	
		Cross-sectional study—Report numbers of outcome events or summary measures	
Main results	16	(*a*) Give unadjusted estimates and, if applicable, confounder-adjusted estimates and their precision (eg, 95% confidence interval). Make clear which confounders were adjusted for and why they were included	10-11, Tables
		(*b*) Report category boundaries when continuous variables were categorized	
		(*c*) If relevant, consider translating estimates of relative risk into absolute risk for a meaningful time period	
Other analyses	17	Report other analyses done—eg analyses of subgroups and interactions, and sensitivity analyses	10-11
**Discussion**			
Key results	18	Summarise key results with reference to study objectives	12
Limitations	19	Discuss limitations of the study, taking into account sources of potential bias or imprecision. Discuss both direction and magnitude of any potential bias	12
Interpretation	20	Give a cautious overall interpretation of results considering objectives, limitations, multiplicity of analyses, results from similar studies, and other relevant evidence	12-14
Generalisability	21	Discuss the generalisability (external validity) of the study results	15
**Other information**			
Funding	22	Give the source of funding and the role of the funders for the present study and, if applicable, for the original study on which the present article is based	15

*Give information separately for cases and controls in case-control studies and, if applicable, for exposed and unexposed groups in cohort and cross-sectional studies.

For peer review only

Note: An Explanation and Elaboration article discusses each checklist item and gives methodological background and published examples of transparent reporting. The STROBE checklist is best used in conjunction with this article (freely available on the Web sites of PLoS Medicine at http://www.plosmedicine.org/, Annals of Internal Medicine at http://www.annals.org/, and Epidemiology at http://www.epidem.com/). Information on the STROBE Initiative is available at www.strobe-statement.org.

For peer review only
